# Multi-omics profiles of the intestinal microbiome in irritable bowel syndrome and its bowel habit subtypes

**DOI:** 10.1186/s40168-022-01450-5

**Published:** 2023-01-10

**Authors:** Jonathan P. Jacobs, Venu Lagishetty, Megan C. Hauer, Jennifer S. Labus, Tien S. Dong, Ryan Toma, Momchilo Vuyisich, Bruce D. Naliboff, Jeffrey M. Lackner, Arpana Gupta, Kirsten Tillisch, Emeran A. Mayer

**Affiliations:** 1 Vatche and Tamar Manoukian Division of Digestive Diseases, Department of Medicine, David Geffen School of Medicine, University of California Los Angeles, Los Angeles, CA USA; 2 G. Oppenheimer Center for Neurobiology of Stress and Resilience, David Geffen School of Medicine, University of California Los Angeles, Los Angeles, CA USA; 3 Division of Gastroenterology, Hepatology and Parenteral Nutrition, VA Greater Los Angeles Healthcare System, Los Angeles, CA USA; 4 Viome Life Sciences, Bellevue, WA USA; 5 Division of Behavioral Medicine, Department of Medicine, Jacobs School of Medicine, University at Buffalo, SUNY, Buffalo, NY USA; 6 Integrative Medicine, VA Greater Los Angeles Healthcare System, Los Angeles, CA USA

**Keywords:** Irritable bowel syndrome, Bowel habit subtypes, Microbiome, Multi-omics, Metatranscriptomics, Metabolomics, Biomarkers

## Abstract

**Background:**

Irritable bowel syndrome (IBS) is a common gastrointestinal disorder that is thought to involve alterations in the gut microbiome, but robust microbial signatures have been challenging to identify. As prior studies have primarily focused on composition, we hypothesized that multi-omics assessment of microbial function incorporating both metatranscriptomics and metabolomics would further delineate microbial profiles of IBS and its subtypes.

**Methods:**

Fecal samples were collected from a racially/ethnically diverse cohort of 495 subjects, including 318 IBS patients and 177 healthy controls, for analysis by 16S rRNA gene sequencing (*n* = 486), metatranscriptomics (*n* = 327), and untargeted metabolomics (*n* = 368). Differentially abundant microbes, predicted genes, transcripts, and metabolites in IBS were identified by multivariate models incorporating age, sex, race/ethnicity, BMI, diet, and HAD-Anxiety. Inter-omic functional relationships were assessed by transcript/gene ratios and microbial metabolic modeling. Differential features were used to construct random forests classifiers.

**Results:**

IBS was associated with global alterations in microbiome composition by 16S rRNA sequencing and metatranscriptomics, and in microbiome function by predicted metagenomics, metatranscriptomics, and metabolomics. After adjusting for age, sex, race/ethnicity, BMI, diet, and anxiety, IBS was associated with differential abundance of bacterial taxa such as *Bacteroides dorei*; metabolites including increased tyramine and decreased gentisate and hydrocinnamate; and transcripts related to fructooligosaccharide and polyol utilization. IBS further showed transcriptional upregulation of enzymes involved in fructose and glucan metabolism as well as the succinate pathway of carbohydrate fermentation. A multi-omics classifier for IBS had significantly higher accuracy (AUC 0.82) than classifiers using individual datasets. Diarrhea-predominant IBS (IBS-D) demonstrated shifts in the metatranscriptome and metabolome including increased bile acids, polyamines, succinate pathway intermediates (malate, fumarate), and transcripts involved in fructose, mannose, and polyol metabolism compared to constipation-predominant IBS (IBS-C). A classifier incorporating metabolites and gene-normalized transcripts differentiated IBS-D from IBS-C with high accuracy (AUC 0.86).

**Conclusions:**

IBS is characterized by a multi-omics microbial signature indicating increased capacity to utilize fermentable carbohydrates—consistent with the clinical benefit of diets restricting this energy source—that also includes multiple previously unrecognized metabolites and metabolic pathways. These findings support the need for integrative assessment of microbial function to investigate the microbiome in IBS and identify novel microbiome-related therapeutic targets.

Video Abstract

**Supplementary Information:**

The online version contains supplementary material available at 10.1186/s40168-022-01450-5.

## Introduction

Irritable bowel syndrome (IBS) is the most common gastrointestinal disorder, affecting 10–15% of the USA population [1]. IBS is currently defined by symptom criteria, including recurrent abdominal pain associated with altered bowel habits in the absence of detectable anatomic, inflammatory, or biochemical pathology [2]. There has been considerable interest in the role of the gut microbiome in IBS. This is supported by the development of IBS symptoms following gastrointestinal infections in some patients, the relationship of IBS symptomatology with diet, and effects of GI microbiota on intestinal motility and visceral sensitivity [3]. Moreover, microbiota transfer from IBS patients into rodents has been reported to confer alterations in intestinal transit and visceral sensitivity to recipient animals [4, 5].

Despite a strong scientific rationale and gastrointestinal symptom improvements following interventions believed to act via the microbiome including rifaximin and FODMAPS diet, existing human microbiome association studies have shown mixed results. Large studies of microbiome composition by 16S rRNA gene sequencing or shotgun metagenomics have in some cases shown minimal diversity change and taxonomic associations with IBS, while others have reported reduced diversity and taxonomic shifts that were not consistent across studies [6–10]. Recent studies have shifted to focus on functional characterization of the microbiome by shotgun metagenomics, which assesses functional potential by microbial gene content, or metabolomics, which assesses products of bacterial metabolism detected in feces [7, 11–16]. While these studies have reported significant shifts in gene content and metabolites, the specific features have not been consistent across studies. Overall, no robust compositional or functional microbiome signature has emerged for IBS diagnosis, unlike other gastrointestinal conditions such as inflammatory bowel disease [17]. This likely reflects variability in clinical definitions of IBS, demographic heterogeneity across studies, confounding factors that influence the microbiome, differences among phenotypic subsets of IBS (with some studies arguing that only certain bowel habit subtypes show differences compared to controls), and challenges of inferring bacterial metabolism from bacterial gene content or fecal metabolite measurements alone.

To provide further insight into the functional properties of gut microbes in IBS, we assembled a diverse, well-phenotyped cross-sectional cohort of 318 IBS patients and 177 healthy controls for multi-omics analysis of the gut microbiome. This encompassed compositional assessment by 16S rRNA gene sequencing and functional assessment by a combination of metatranscriptomics and fecal metabolomics. We found that IBS could be robustly distinguished from healthy controls by a functional signature derived from differential bacterial transcripts and microbiome-associated metabolites after adjusting for multiple covariates influencing the microbiome, and that IBS bowel habit subtypes were similarly distinguished by multi-omics functional signatures.

## Methods

### Participants

Two hundred seventy-five IBS patients and 177 healthy controls were recruited from the Los Angeles area by community advertisement and an additional 43 IBS patients were recruited from the Buffalo area for a study of cognitive behavioral therapy [18]. IBS patients were eligible if they were over age 18 and diagnosed with IBS via Rome III criteria [19]. Potential participants were excluded if they had accompanying organic GI disease that could contribute to presenting IBS symptoms, malignancy in the past 5 years, body mass index (BMI) greater than 35, reported a gastrointestinal infection within 2 weeks before evaluation, or used a gut-sensitive antibiotic during the 12 weeks prior to evaluation. Participants were asked to provide a fecal sample and complete demographic and medical questionnaires. Dietary pattern was assessed using our previously validated dietary pattern form [20]. Psychological and symptom instruments included Hospital Anxiety and Depression (HAD), State-Trait Anxiety Inventory (STAI), Visceral Sensitivity Index (VSI)—a measure of gastrointestinal symptom-specific anxiety, and Pennebaker Inventory of Limbic Languidness (PILL)—an assessment of frequency of stress-sensitive somatic complaints. The IBS Symptom Severity Scale (IBS-SSS) was used to measure IBS symptom severity including pain, distention, bowel dysfunction, and quality of life [21]. This study was approved by the Institutional Review Boards at UCLA and the University at Buffalo.

### Fresh frozen fecal sample collection

Participants were provided with home stool collection kits and asked to store their stool samples in a freezer immediately after collection until they could either be dropped off to a study coordinator or picked up by a courier service within 24–48 h of collection. Study coordinators then transferred samples to lab freezers for long-term storage at − 80°C. Frozen fecal samples were later ground into a coarse powder by mortar and pestle under liquid nitrogen then aliquoted.

### Fecal 16S rRNA gene sequencing

DNA extraction by bead beating and amplification of the V4 hypervariable region of the 16S rRNA gene were performed according to our published protocol [22, 23]. Sequencing libraries underwent 2 × 150 or 2 × 250 sequencing on Illumina HiSeq 2500 to a mean depth of 67,801 merged sequences per sample. Samples in each of two sequencing batches (292 and 195 samples) were separately processed using the DADA2 R package to generate tables of denoised amplicon sequence variants (ASVs) which were then merged [24]. Taxonomy was assigned to the 11,429 ASVs using the SILVA 138.1 database. The final ASV table was inputted into PICRUSt2 to predict abundances of bacterial gene families annotated as KEGG orthologs (KOs) based on nearest reference genomes to 16S sequences [25, 26].

### Fecal metatranscriptomics

Fecal aliquots were sent to Viome Life Sciences, Inc., for RNA extraction, metatranscriptomics sequencing, and annotation as previously described [27]. In brief, RNA extraction by bead beading was performed, DNA degraded by DNase, and 16S/23S ribosomal RNA depleted by subtractive hybridization. Sequencing libraries were prepared from the resulting RNA and underwent 150 × 2 paired-end sequencing on Illumina NovaSeq. Taxonomy was assigned by aligning sequencing reads to a precomputed database of unique k-mers; 898 taxa were identified, including bacteria, fungi, viruses, and bacteriophages. Functional annotation was performed by aligning sequencing reads to the integrated gene catalog from the MetaHIT consortium then mapping these genes to the KEGG database; 5896 distinct transcripts annotated as KOs were identified [28].

### Fecal metabolomics

Fecal aliquots were shipped to Metabolon, Inc., for analysis on their global HD4 metabolomics platform. Samples were run in three batches; the largest contained 250 samples and additional batches included 75 and 43 samples. Compounds were identified by comparison of spectral features to Metabolon’s proprietary library, which includes MS/MS spectral data on more than 3300 purified standards. Within each batch, features were retained that were detected in over 30% of samples. Imputation of missing data was performed separately for each batch using a k-nearest neighbor algorithm (kNN, *k* = 10) as previously described for Metabolon datasets [29]. The three batches were then combined using reference-based ComBat implemented in the sva package in R with the largest batch used as a reference and IBS vs. HC as a covariate; only the 601 metabolites with greater than 30% detection rate in all three batches were retained [30–32]. Metabolomics data subsequently underwent log2 transformation and normalization by the vsn2 package in R prior to downstream analyses [33].

### Alpha and beta diversity analyses

Microbial alpha diversity was assessed on unfiltered 16S data (rarefied to 10,000 sequences/sample) and metatranscriptomics taxonomy data using the Shannon index of richness and evenness calculated with the phyloseq package in R [34]. Significance was assessed by the Mann-Whitney *U* test and multivariate ANOVA using the aov function in R with sex, age, race, BMI, diet category, and HAD-Anxiety as covariates. Beta diversity analysis was performed on all five datasets including 16S composition, predicted metagenome, metatranscriptome taxonomy, metatranscriptome KO, and metabolome. Bray-Curtis dissimilarity was applied for all datasets except metabolomics, for which Euclidean distance was used since it is not a compositional dataset. In each case, datasets were filtered to remove features present in less than 10% of samples, leaving 228 ASVs, 5370 predicted genes, 269 metatranscriptomics taxa, 3837 transcripts, and 601 metabolites. Constrained ordination of these distance matrices was performed using distance-based redundancy analysis (dbRDA) implemented with the capscale function of vegan in R [35]. Models for dbRDA included sex, age, race/ethnicity (African-American, Asian, Hispanic, non-Hispanic white, multiracial), BMI, diet category (standard, restrictive, other), HAD-Anxiety, batch (sequencing or metabolomics), and IBS group. Missing HAD and dietary pattern data were imputed by kNN using the VIM package in R for use as covariates in these and other multivariate models. Visualization of categorical and continuous variables on the first two constrained axes of dbRDA ordination was performed using the envfit function of vegan. The significance of differences in beta diversity was assessed using permutational multivariate analysis of variance (PERMANOVA) implemented with the adonis2 function of vegan with 10,000 permutations [36]. Univariate models included IBS group or one of the covariates and batch (sequencing or metabolomics); multivariate models also included sex, age, race, BMI, diet category, and HAD-Anxiety. Adonis2 was run by “margin,” which calculates the marginal *R*^2^ for each variable after adjusting for the other variables in the model (i.e., running separate models for each variable in which it is ordered last).

### Differential abundance testing

Differentially abundant microbes, genes, transcripts, and metabolites were identified using multivariate general linear models implemented in MaAsLin2 [37]. Sequencing data underwent total sum scaling to generate relative abundances which were log-transformed prior to model fitting. Normalized, log-transformed metabolomics data did not undergo further transformation. All datasets were filtered to retain features present in > 10% of samples. *P* alues were adjusted for multiple hypothesis testing by the Benjamini-Hochberg method to generate *q* values. Significance was set at *q* < 0.25 for all analyses as is recommended for MaAsLin2 [37]. Functional annotations were made using KEGG pathway assignments and metabolite categories provided by Metabolon.

### Inter-omic comparisons

Global comparison of datasets was performed by applying the Mantel test to distance matrices of Bray-Curtis dissimilarity (or Euclidean distance for metabolomics) using vegan in R. The resulting Mantel r was calculated using Spearman correlation, with significance determined by 10,000 permutations. Visualization of global similarities across datasets was performed by rotating then superimposing one dbRDA ordination onto another using Procrustes in vegan.

Comparison of RNA vs. DNA taxonomic representation was performed at the genus level to maximize matching of taxa between the metatranscriptome and 16S data given the limitations of taxonomic resolution with 16S rRNA gene sequencing. For each genus, ratios of relative abundances were calculated for samples in which there was non-zero abundance in both the 16S and metatranscriptomics data; if a genus was not detected in one dataset, no ratio was calculated. The same strategy was used to calculate RNA/DNA ratios comparing transcript relative abundances to predicted gene relative abundances. RNA/DNA ratios for genera and KOs were only further analyzed if the features were detected in > 25% of both DNA and RNA samples. Log-transformed ratios were analyzed by multivariate general linear models in MaAsLin2 to identify statistically significant differences (*q* < 0.25) between IBS and HC in RNA/DNA ratios after adjusting for covariates. This approach has previously been applied to simulated and real fecal datasets and shown to be suitable for differential expression analysis [38]. Missing values were dropped for model fitting in MaAsLin2 and imputed by kNN for input into random forests classifiers.

Pathway enrichment analysis for differentially expressed KOs was performed using Gene Set Enrichment Analysis (GSEA) software [39]. *q* values were used to rank KOs by their strength of association with IBS or IBS bowel habits, and KO assignments to KEGG pathways were inputted as gene sets. Significance was calculated using the classic enrichment statistic, 10,000 permutations, and a threshold of *p* < 0.05.

### Identification of metabolites associated with microbial metabolism

Metabolic modeling was performed using MIMOSA2 to estimate the community-wide metabolic output of the metatranscriptome and predicted metagenome as summarized by Community-wide Metabolic Potential (CMP) scores [40, 41]. The scores are a surrogate for the relative capacity of the metagenome or metatranscriptome to produce or deplete the metabolite. MIMOSA2 performs regression of CMP scores for each metabolite with measured levels of that metabolite to identify a subset of metabolites with significant positive association with microbial metabolic potential. The recommended threshold of *p* < 0.10 was used.

### Random forests classifiers

Datasets were divided 60%/40% into training and test sets with equivalent proportion of IBS subjects (or BH subtypes) and random forests classifiers were constructed using the caret R package [42]. Differentially abundant features were inputted into the classifiers. Contribution of each feature to classifier accuracy was assessed by variable importance scores, which represent the decrease in classifier accuracy when that variable is permuted. Features were retained in the final model if they had an importance score > 2 in the initial model iteration using training data. The accuracy of the resulting classifiers was determined by calculating the area under the receiver operating characteristic curve (AUC) using test data. Significance of differences in classifier performance was assessed using the bootstrap method of roc.test in the pROC R package.

## Results

### IBS is associated with shifts in microbial taxa, transcripts, and metabolites

Four hundred ninety-five subjects, including 318 IBS patients and 177 healthy controls (HC), provided fecal samples and completed demographic, dietary, and psychological questionnaires (Table 1). IBS patients and HC did not differ by age (median 30 vs. 29 years old) but a higher proportion of IBS subjects were females (77% vs. 60%). The cohort was racially diverse, with 56% of subjects belonging to a racial/ethnic minority group in the USA. The IBS subjects and HC showed different racial/ethnic composition, with the IBS group having increased proportion of non-Hispanic whites and corresponding reduced proportions of other racial/ethnic groups. There was no statistically significant difference in educational attainment between the two groups, with the majority of subjects being college graduates. Dietary patterns were grouped into one of three categories using a paradigm recently established by our group for IBS studies: standard diet, restrictive diet (which includes gluten-free, lactose-free, and FODMAPS diets), and other (representing dietary patterns considered exclusionary but not in one of the IBS-associated restrictive dietary patterns) [20]. Consistent with our prior findings, IBS patients had increased frequency of restrictive diets—specifically, gluten-free and lactose-free diets (15% vs. 3% and 13% vs. 4%, respectively)—and a corresponding reduction in the frequency of a Standard or Modified American diet (Additional file 1: Table S1). Participants also completed psychological questionnaires including the HAD and STAI, which together measure depression, current anxiety, and trait anxiety, respectively. All three instruments showed elevated scores in IBS compared to HC. As anticipated, IBS patients showed markedly higher scores for visceral sensitivity (VSI) and stress-sensitive somatic complaints (PILL). Approximately equal numbers of IBS patients were diarrhea-predominant (IBS-D, 38%) and constipation-predominant (IBS-C, 35%), and most had moderate (IBS-SSS of 175–300, 49%) or severe (IBS-SSS of 301–500, 27%) symptom severity.

**Table 1:** Cohort demographics

	HC (*N* = 177)	IBS (*N* = 318)	*P* value
Samples			
16S rRNA sequencing	174	312	
Metatranscriptomics	119	208	
Metabolomics	139	229	
Gender			
Male	40%	23%	**0.00013**
Female	60%	77%	
Age	30 (23–38)	29 (23–41)	0.57
BMI	26.9 (23.4–30.2)	23.4 (21.0–26.0)	**3 × 10**^**−12**^
Race/ethnicity			
Non-Hispanic white	31%	52%	**0.00016**
Hispanic	25%	20%	
African-American	12%	7%	
Asian	21%	14%	
Multiracial	10%	7%	
Education			
Some high school	1%	1%	0.12
High school grad	7%	6%	
Some college	28%	39%	
College grad	33%	24%	
Any postgrad	31%	30%	
Diet			
Standard	79%	55%	**0.0005**
Restrictive	8%	30%	
Other	13%	15%	
HAD-Anxiety	3 (1–6)	7 (4–10)	**8 × 10**^**−14**^
HAD-Depression	1 (0–3)	2 (1–5)	**7 × 10**^**−6**^
STAI Trait Anxiety	32 (26–38)	37 (27–44)	**0.0055**
PILL	4 (2–7)	16 (11–22)	**< 2 × 10**^**−16**^
VSI	2 (0–8)	40 (28–54)	**< 2 × 10**^**−16**^
Bowel habit subtype			
IBS-D		38%	
IBS-C		35%	
IBS-M		17%	
IBS-U		9%	
IBS-SSS		241 (184–312)	

Values shown as median (interquartile range); *p* values calculated by the Mann-Whitney *U* test (numerical traits) or Fisher’s exact test (categorical traits)

**Fig. 1:**
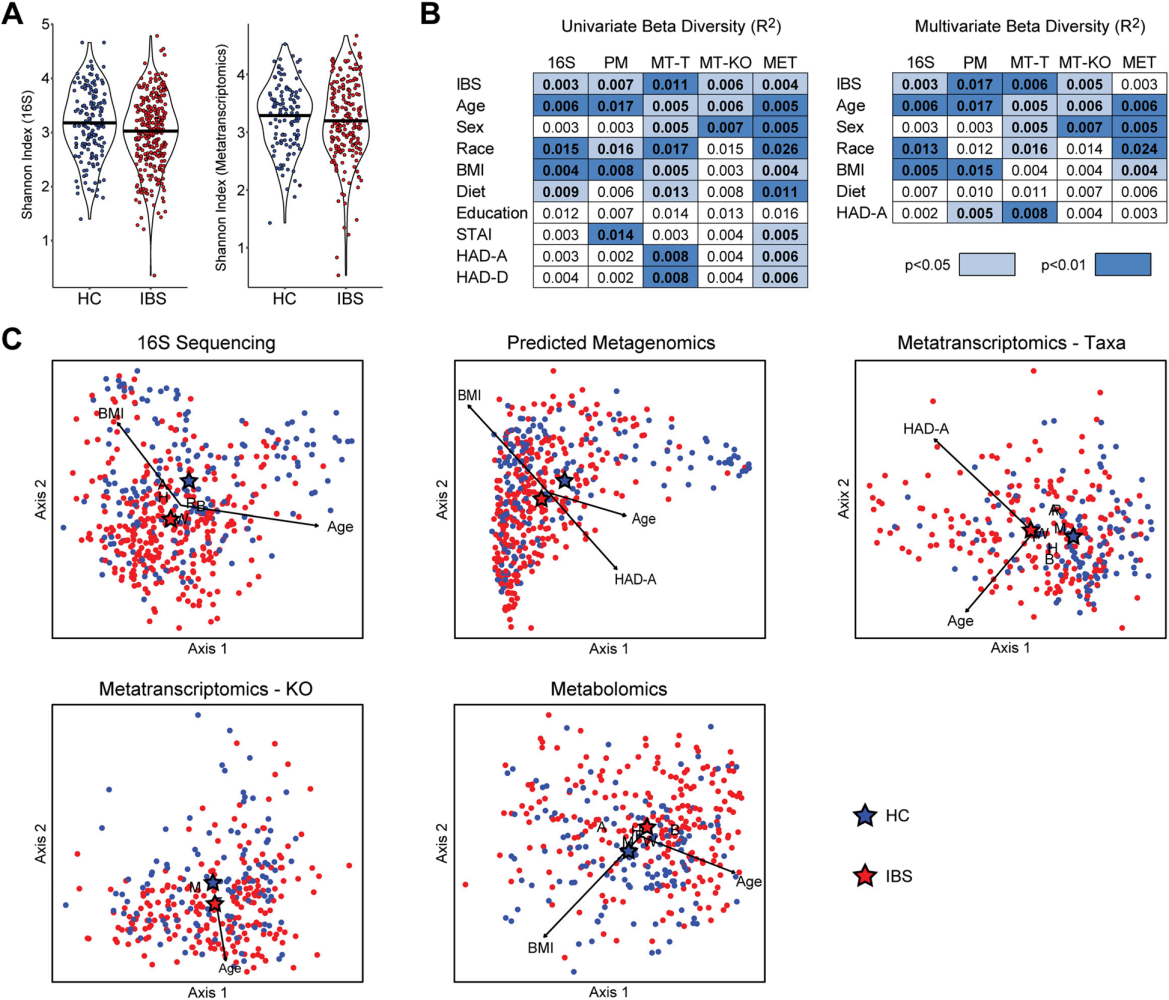
IBS is associated with global alterations in microbiome composition and function. **A** The Shannon index of microbial richness and evenness in fecal samples from IBS subjects and healthy controls (HC) is shown for 16S rRNA sequencing and metatranscriptomics data. **B** Beta diversity was assessed by Bray-Curtis dissimilarity for 16S rRNA sequence data, predicted metagenomics (PM), metatranscriptomics taxonomy (MT-T), and KEGG orthology transcript annotations (MT-KO). Euclidean distance was used for normalized metabolomics data (MET). Contribution of clinical and demographic traits to variation in these five datasets was determined by *R*^2^ calculated from univariate (left) and multivariate (right) PERMANOVA. *R*^2^ in the multivariate model reflects the remaining explained variance after accounting for the other variables. All PERMANOVA analyses included batch (sequencing or metabolomics) as a covariate. Significance of differences was calculated by permutation and is denoted by color. (**C**) Distance-based redundancy analysis (dbRDA) was performed to visualize variation in beta diversity related to IBS status, age, sex, race/ethnicity, BMI, dietary category, and HAD-Anxiety (HAD-A). IBS group and statistically significant categorical variables are denoted by letters or symbols indicating the centroid for each category. Statistically significant continuous variables are shown as arrows originating from the centroid of all samples, with length proportional to strength of association. F = female, M = male, A = Asian, B = African-American, H = Hispanic, W = non-Hispanic white, R = multiracial

Fecal samples underwent analysis by 16S rRNA gene sequencing (*n* = 486), metatranscriptomics sequencing (*n* = 327), and untargeted global metabolomics (*n* = 368). Microbial composition was assessed by 16S rRNA gene abundances and by taxonomic assignment of sequenced transcripts, which reflects composition weighted by transcriptional activity. Alpha diversity (i.e., within-sample diversity) was compared between IBS and HC using the Shannon index of richness and evenness. Both compositional datasets showed no significant difference in Shannon index by non-parametric tests and multivariate linear models (Fig. 1A). In contrast, beta diversity analysis (i.e., diversity across samples) using Bray-Curtis dissimilarity demonstrated statistically significant differences in composition by 16S rRNA profiles and metatranscriptomics taxonomy between IBS and HC (Fig. 1B). Many covariates were also found to be significantly associated with microbial composition in one or both datasets, including age, sex, race, BMI, dietary category, HAD-Anxiety, and HAD-Depression. Given the strong correlation between anxiety and depression and the greater differential between IBS and HC in HAD-Anxiety, this was selected as a covariate to represent the association of mood with the microbiome. In multivariate analyses adjusting for these covariates, IBS remained significantly associated with microbial composition by 16S rRNA and metatranscriptomics sequencing, as did several of the covariates including age and race (Fig. 1B, C).

**Fig. 2:**
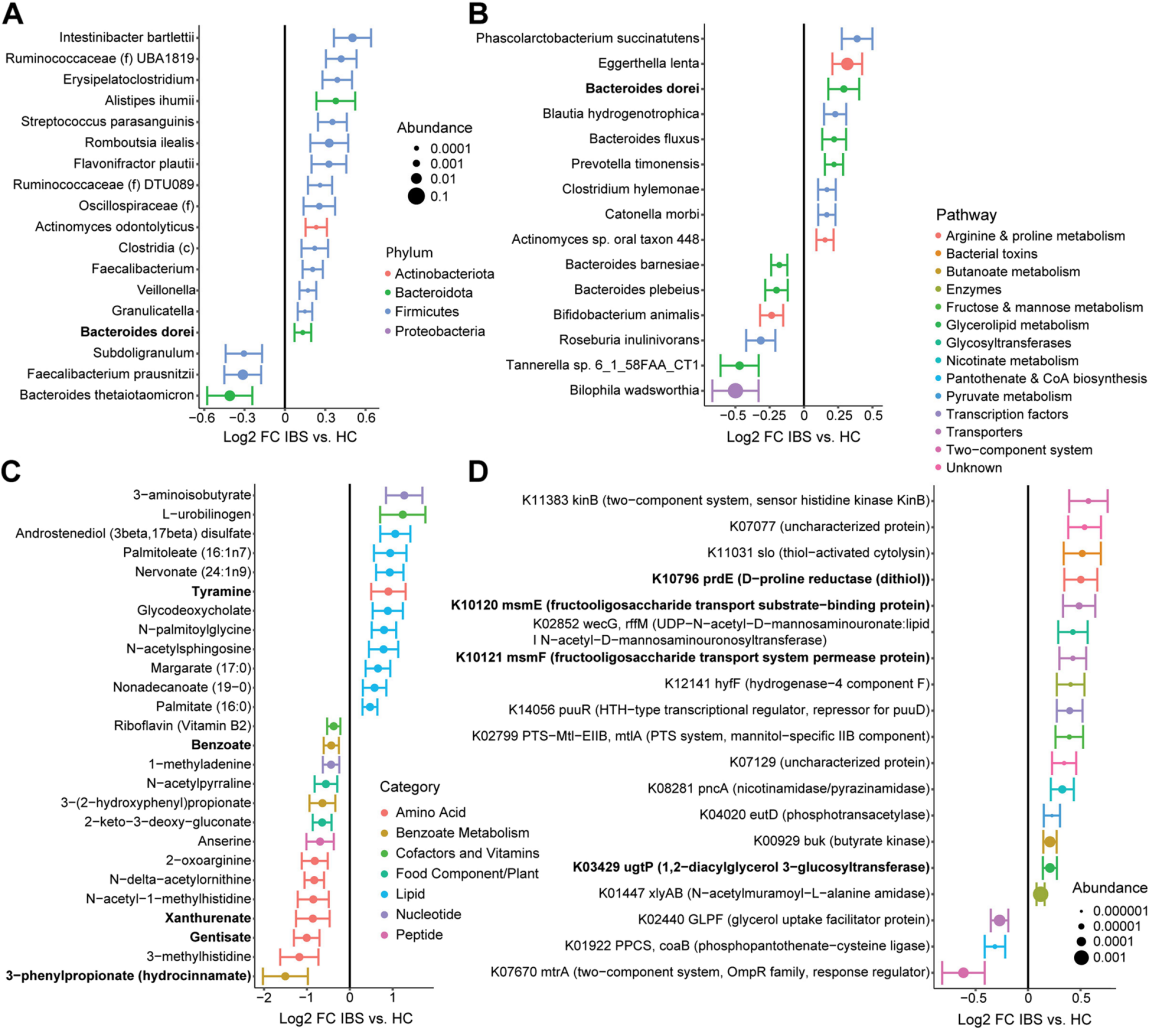
IBS is characterized by altered abundances of bacterial taxa, metabolites, and transcripts, including for genes involved in fructooligosaccharide utilization. **A**, **B** Differentially abundant bacterial taxa (*q* < 0.25) between IBS subjects and HC were identified in multivariate models adjusting for batch, age, sex, race/ethnicity, BMI, dietary category, and HAD-A. Results are shown for **A** 16S rRNA sequence data (*n* = 486) and **B** metatranscriptomics data (*n* = 327), with bold indicating the single overlapping taxon (*B. dorei*). Effect size is represented as log2 of the fold change (FC). Color indicates phylum and dot size is proportional to taxon abundance. Bars indicate standard error of log2 fold change estimates. **C** Differentially abundant fecal metabolites (*q* < 0.25 in multivariate models) detected by global untargeted metabolomics (*n* = 368) are shown, with color representing functional category. Bold indicates metabolites that were associated with microbial community metabolic potential by MIMOSA2. **D** Differentially abundant bacterial transcripts, annotated by KEGG KO number, gene symbol, and gene name. Transcripts for genes that were also differentially abundant in the predicted metagenome are shown in bold. Dot size is proportional to transcript relative abundance and color represents KEGG pathway annotation (legend above the plot)

Multivariate general linear models were then used to identify microbial taxa that significantly differed in IBS compared to HC after adjusting for covariates identified by beta diversity analysis as influencing the microbiome including age, sex, race, BMI, diet, and HAD-Anxiety. IBS was found to be characterized by increased abundance of *Alistipes ihumii*, *Bacteroides dorei*, *Actinomyces odontolyticus*, and multiple members of the Firmicutes phylum such as *Intestinibacter bartlettii* and *Roumboutsia ilealis* (Fig. 2A). IBS showed reduced abundance of *Facealibacterium prausnitzii* and *Bacteroides thetaiotamicron*. When assessing the taxonomic profiles of the metatranscriptome, only *B. dorei* showed concordant change with the 16S sequencing findings (Fig. 2B). Bacteria with increased transcript abundance in IBS included *Eggerthella lenta*, two *Bacteroides* species (*B. dorei* and *B. fluxus*), *Phascolarctobacterium succinatutens*, *Blautia hydrogenotrophica*, *Prevotella timonsensis*, *Clostridium hylemonae*, *Catonella morbi*, and an unidentified *Actinomyces* species. IBS showed decreased transcript abundance of *Bilophila wadsworthia, Roseburia inulinivorans*, *Bifidobacterium animals*, and two *Bacteroides* species (*B. plebeius* and *B. barnesiae*).

Microbial function was assessed by bacterial transcript abundances (annotated by KEGG orthology (KO)), bacterial gene abundances predicted from 16S rRNA compositional data using phylogenetically-nearest reference genomes (predicted metagenome), and metabolite levels. IBS status was significantly associated with variation in beta diversity of all three datasets in univariate analyses (Fig. 1B). Among the covariates, age, sex, race, BMI, dietary category, and HAD-Anxiety were significantly associated with the metatranscriptome and metabolome; age, race, and BMI also were significantly associated with the predicted metagenome. After adjustment for these six covariates, IBS remained significantly associated with the metatranscriptome and predicted metagenome but no longer had a significant association with the metabolome (Fig. 1B, C).

Differential abundance testing identified 16 bacterial transcripts with increased abundance in IBS and 3 with decreased abundance (Fig. 2D). Similar analysis was performed to identify differential genes from the predicted metagenome, but given the potential inaccuracies at the KO level of predicted abundances, these were used only to highlight differential KOs found in the metatranscriptomics data [25]. A total of four transcripts showed concordant changes in the predicted metagenome, including increased abundance of D-proline reductase, 1-2-diacylglycerol 3-glucosyltransferase, and two components of the fructooligosaccharide transport system (permease and substrate-binding protein). This suggests that increased levels of these transcripts could be attributable to differences in abundance of their corresponding genes in the metagenome. Other notable shifts in the metatranscriptome included increased abundance of butyrate kinase and the phosphotransferase system for uptake of mannitol (a fermentable polyol).

While IBS was not associated with significant global change in the metabolome after adjusting for covariates, IBS showed statistically significant increases in 12 metabolites (Fig. 2C). Nine of these were classified as lipids including free fatty acids such as palmitate and margarate, a bile acid (glycodeoxycholate), and N-acetylsphingosine. The other elevated metabolites included L-urobilinogen (a product of bacterial metabolism of biliruben), 3-aminoisobutyrate, and tyramine. There were 14 metabolites with reduced levels in IBS, which included 6 amino acid derivatives (e.g., 3-methylhistidine, N-acetyl-1-methylhistidine, gentisate, xanthurenate), anserine (a dipeptide that includes 3-methylhistidine), benzoate as well as two related metabolites (3-(2-hydroxyphenol)propionate and hydrocinnamate), and riboflavin.

### Inter-omic analyses reveal consistent metabolic shifts across datasets that differentiate IBS from HC

Given that all five datasets demonstrated alterations in IBS compared to HC, we then assessed whether there were conserved compositional and functional shifts across multiple data types. Global correlations of datasets were assessed by the Mantel test. All pairwise combinations of the five datasets showed statistically significant positive correlation, with the greatest association (other than 16S and the metagenome predicted from 16S) observed for metatranscriptomics and the metabolome (Additional file 1: Figure S1). These significant associations across datasets were visualized by Procrustes, which highlighted the separation of IBS from HC in superimposed datasets.

**Fig. 3:**
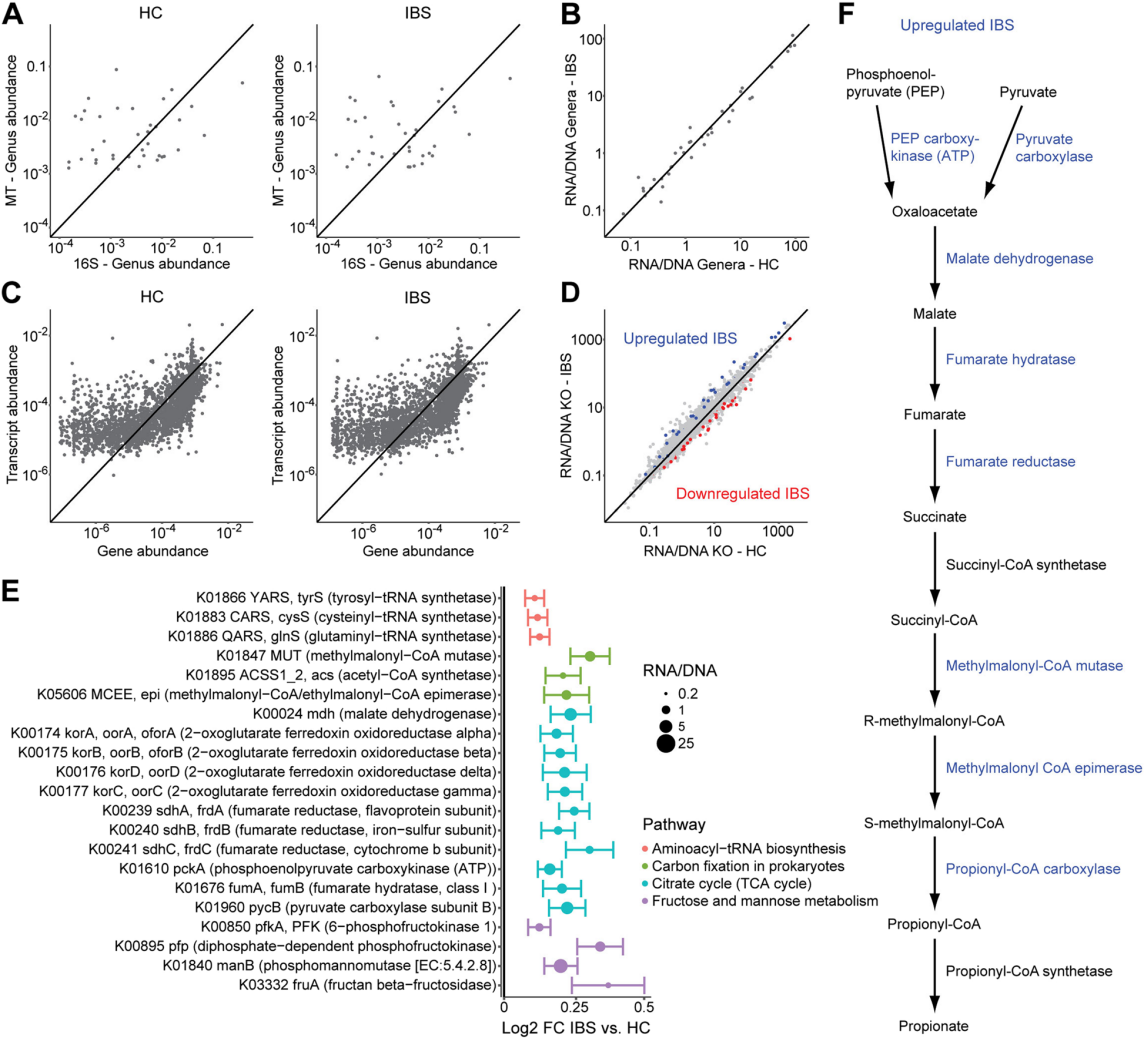
Inter-omic comparison of bacterial taxonomy and function demonstrated upregulation in IBS of transcripts involved in the succinate pathway of carbohydrate fermentation. **A** Scatterplots depicting median relative abundances of genera in 16S rRNA sequence data and in the metatranscriptome for the 322 subjects with both data types available. **B** Median ratios of genus abundances in the metatranscriptome (RNA) vs. 16S data (DNA) are plotted for IBS subjects and HC. **C** Scatterplots of median gene abundances in the predicted metagenome compared to median transcript abundances. **D** Median transcript/gene abundance ratios are plotted for IBS subjects and HC. Transcripts that were significantly upregulated or downregulated in IBS compared to HC (adjusting for batch, age, sex, race/ethnicity, BMI, dietary category, and HAD-A) are colored. **E** Pathways that were significantly enriched in differentially regulated transcripts were identified. Significant transcripts within these pathways (represented by dot color) are shown, with dot size proportional to RNA/DNA ratio. Bars indicate standard error of log2 fold change estimates. **F** Succinate pathway of carbohydrate fermentation. Enzymes transcriptionally upregulated in IBS are colored blue

We further investigated three categories of inter-omic association. First, we assessed the ratio of microbial abundances in the metatranscriptome (RNA) to those from 16S sequencing (DNA). These ratios broadly represent transcriptional activity of microbes [43]. This analysis was performed at the genus level to facilitate matching of taxonomic assignments from 16S sequencing (which does not consistently achieve species resolution) with those from metatranscriptomics. Consistent with prior reports, transcriptional representation of microbes could differ by orders of magnitude compared to their abundance in the metagenome, differentiating microbes that are transcriptionally active from those that are quiescent (Fig. 3A). The most transcriptionally active genus was *Veillonella* (median RNA/DNA 89 in HC and 114 in IBS) and the least transcriptionally active genus was *Faecalibacterium* (median RNA/DNA 0.074 in HC and 0.087 in IBS). Comparing IBS to HC, the two groups were found to have similar patterns of microbial transcriptional activity at the genus level (Fig. 3B). No taxa showed a statistically significant difference between IBS and HC.

Second, we calculated gene-normalized transcript abundances using the ratio of transcript abundances (RNA) to predicted gene abundances (DNA) for differential gene expression analysis. This approach provides an assessment of transcriptional upregulation or downregulation, which is not possible with transcript abundances alone as these reflect a combination of gene abundance as well as transcriptional regulation. Transcripts were found to have a wide range of ratios relative to genes, from less than 0.1 to over 1000 (Fig. 3C). While RNA/DNA ratios were highly correlated between IBS and HC, 182 KOs were found to be differentially regulated between IBS and HC after adjusting for covariates (Fig. 3D, Additional file 1: Table S2). To identify consistent functional shifts among gene-normalized transcript abundances, pathway enrichment analysis was performed. This demonstrated that the pathways of citrate cycle, fructose and mannose metabolism, carbon fixation in prokaryotes, and aminoacyl-tRNA biosynthesis were enriched in differentially regulated transcripts (Fig. 3E). Within these pathways, 21 differential transcripts were highlighted which were all upregulated in IBS. This included multiple key enzymes in the citric acid cycle including pyruvate carboxylase, phosphoenolpyruvate carboxylase, succinate dehydrogenase, fumarate hydratase, and malate dehydrogenase. The carbon fixation pathway transcripts included acetyl-CoA synthetase as well as methylmalonyl-CoA mutase and methylmalonyl-CoA epimerase (which are involved in the production of the short chain fatty acid, propionate) [44]. Taken together, the transcripts in these two pathways encompass nearly all of the enzymes in the succinate pathway for carbohydrate fermentation to propionate (Fig. 3F) [45]. There were also three transcripts for subunits of the 2-oxoglutarate ferredoxin oxidoreductase, which fixes carbon in the reductive tricarboxylic acid cycle by adding CO_2_ to succinyl-CoA to generate 2-oxoglutarate. The fructose- and mannose-related metabolism transcripts included fructan beta-fructosidase (breakdown of fructans such as inulin), 6-phosphofructokinase 1 (entry of fructose into the glycolytic pathway), diphosphate-dependent phosphofructokinase, and phosphomannomutase. Three tRNA synthetases (glutaminyl, cysteinyl, tryosyl) were also upregulated, though with low magnitude of effect compared to the other transcripts.

Third, we identified fecal metabolites that were likely influenced by microbial metabolism by modeling microbial metabolic potential from metatranscriptomics and predicted metagenomics. Using this strategy, 5 differential metabolites were associated with the microbiome (Additional file 1: Figure S2). Of these, only one metabolite had increased levels in IBS–tyramine, which was associated with metabolic potential from the metatranscriptome. Of the four decreased metabolites, hydrocinnamate was found to be significantly associated with microbial metabolic potential by both metatranscriptomics and predicted metagenomics. Benzoate and gentisate were associated with the predicted metagenome and xanthurenate with the metatranscriptome.

**Fig. 4:**
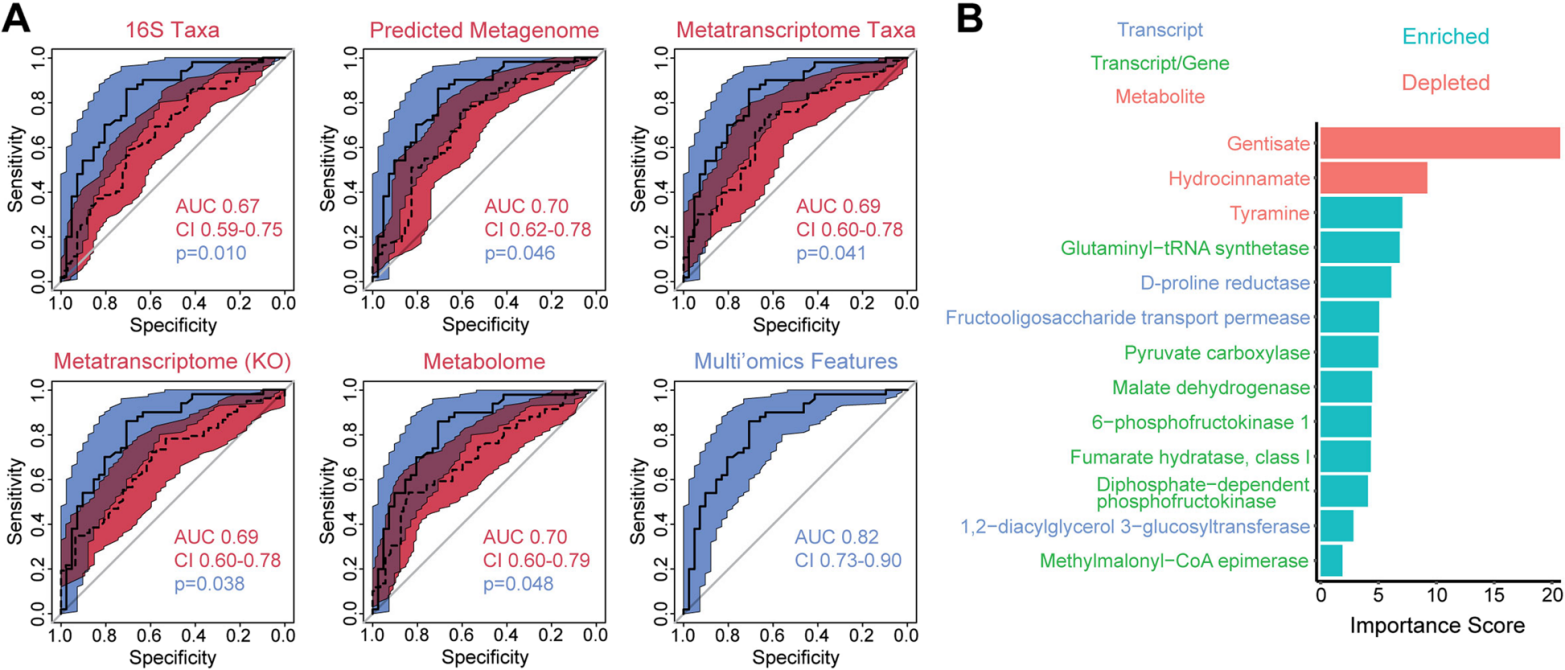
Multi-omics microbiome classifier for IBS showed increased accuracy compared to classifiers using single datasets. **A** ROC curves for random forest classifiers constructed from differentially abundant features in each of the five datasets (colored in red) are compared to the ROC curve for a multi-omics classifier (colored in blue). The multi-omics classifier was constructed from transcripts that were differentially abundant in both the metatranscriptome and predicted metagenome, significantly upregulated transcripts in enriched pathways, and differentially abundant metabolites associated with microbial community metabolic potential. All classifiers were trained on 60% of the dataset and tested on the remaining 40% of samples (*n* = 230 with all three data types). Colored areas indicate the 95% confidence intervals of the ROC curves. *P* values for the AUC of single dataset classifiers compared to the multi-omics classifier were calculated by bootstrapping. **B** Importance scores are shown for features included in the multi-omics classifier, colored by feature type. Bar color indicates whether each feature shown was enriched or depleted in IBS subjects compared to HC

These three sets of inter-omic analyses yielded a group of features associated with IBS based upon analyses incorporating two datasets. This included differentially abundant gene-normalized transcripts in enriched pathways, transcripts that were differentially abundant in both the metatranscriptome and predicted metagenome, and differentially abundant metabolites that were associated with microbial metabolic potential. Using these multi-omics features, a random forests classifier was trained to differentiate IBS from HC that had AUC of 0.82. This performance was statistically significantly superior to classifiers constructed using microbial features from single datasets, which achieved AUC of 0.67–0.70 (Fig. 4). The three microbial features that contributed most to this classifier were metabolites: gentisate, hydrocinnamate, and tyramine. The other ten features in the classifier included three transcripts related to fructooligosaccharide metabolism (diphosphate-dependent phosphofructokinase, 6-phosphofructokinase 1, fructooligosaccharide transport permease protein), three transcripts in the citric acid cycle (pyruvate carboxylase, malate dehydrogenase, fumarate hydratase), glutaminyl-tRNA synthetase, D-proline reductase, 1,2-diacylglycerol 3-glucosyltransferase, and methylmalonyl-CoA epimerase.

### Bacterial transcripts and metabolites differentiate IBS bowel habit subtypes

**Fig. 5:**
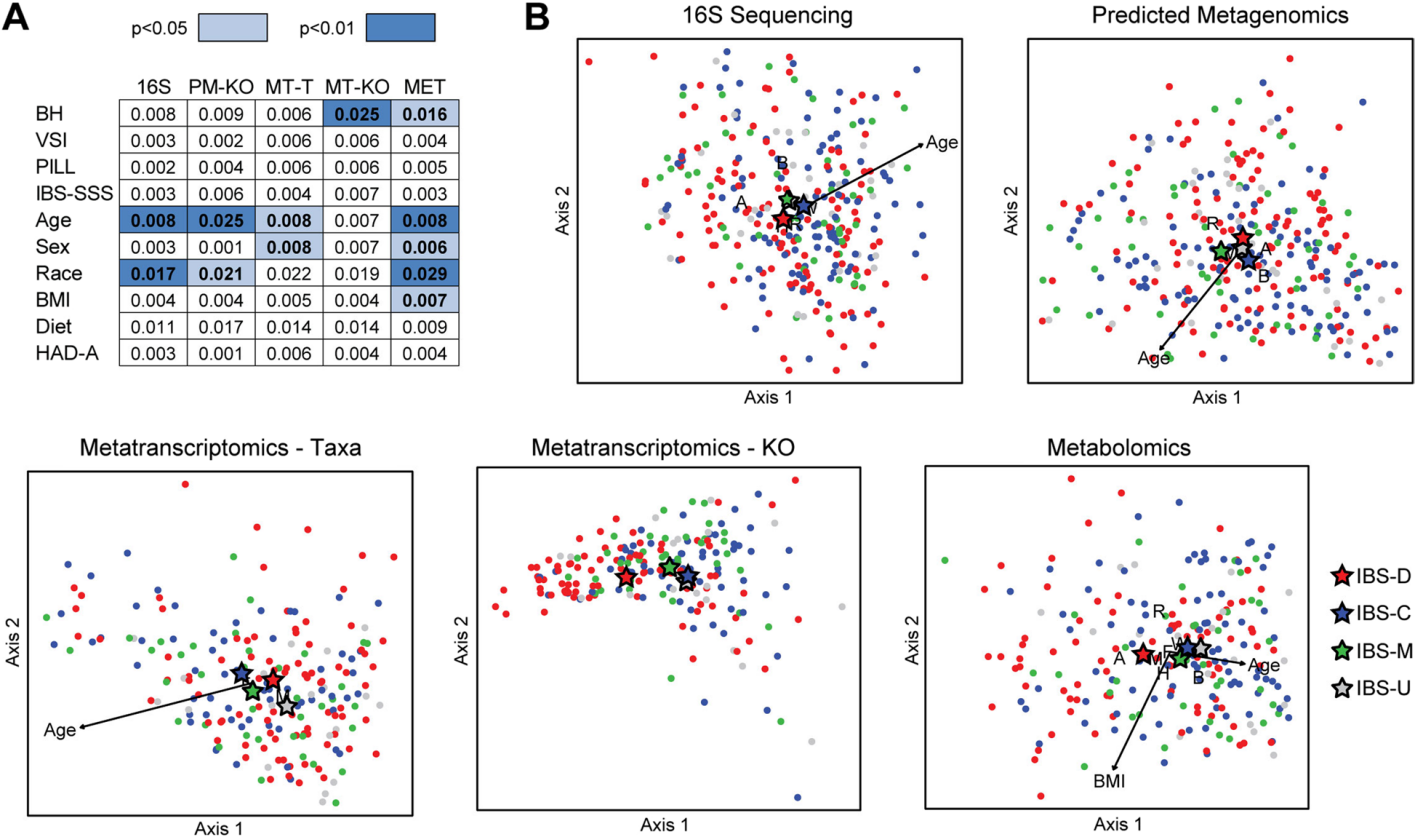
IBS bowel habit (BH) subtypes have distinct functional profiles by metatranscriptomics and metabolomics. **A** Multivariate PERMONOVA models were used to assess the association of phenotypes within IBS including BH subtype, visceral sensitivity (VSI), general physical symptom perception (PILL), and IBS severity (IBS-SSS) with the five datasets, adjusting for batch, age, sex, race/ethnicity, BMI, dietary category, and HAD-A. **B** DbRDA plots for each of the five datasets visualizing differences in beta diversity related to BH subtype and significant categorical or continuous covariates. F = female, M = male, A = Asian, B = African-American, H = Hispanic, W = non-Hispanic white, R = multiracial

Having identified robust microbial profiles differentiating IBS from HC, we then assessed the relationship of microbial composition and function to phenotypes within IBS. Bowel habit subtypes were significantly associated with metatranscriptomics KOs and metabolomics but not with microbiome composition or the predicted metagenome (Fig. 5). Visceral sensitivity (VSI), general physical symptom perception (PILL), and IBS-specific symptom severity (IBS-SSS) were not significantly associated with either global microbiome composition or function by transcripts, predicted genes, and metabolites.

To further delineate microbial functional features that were associated with IBS bowel habit subtype, we focused on comparison of the diarrhea-predominant (IBS-D) and constipation-predominant IBS-C) subtypes. These are the two most numerous BH subtypes and show more consistent BH patterns compared to the remaining two subtypes (IBS-mixed, IBS-M; IBS-unspecified, IBS-U). Differential abundance testing further supported the absence of a clear taxonomic signature of IBS-D vs. IBS-C, as only 2 taxa were significantly different by 16S sequencing and 4 taxa by metatranscriptomics with no overlap between the two datasets (Additional file 1: Figure S3A). In contrast, metatranscriptomics functional assessment demonstrated 54 differential transcripts belonging to diverse pathways, included 51 increased in IBS-D (Additional file 1: Figure S3B). Among these, many were associated with fructose and mannose metabolism pathway, including L-iditol 2-dehydrogenase (polyol dehydrogenase), three components of the mannose-specific phosphotransferase system, and L-fucose/D-arabinose isomerase (which produces ribulose). There was also enrichment of transcripts involved in D-glutamate synthesis (glutamate synthase and glutamate racemase) and ethanolamine utilization. None of these overlapped with differential predicted gene abundances.

**Fig. 6:**
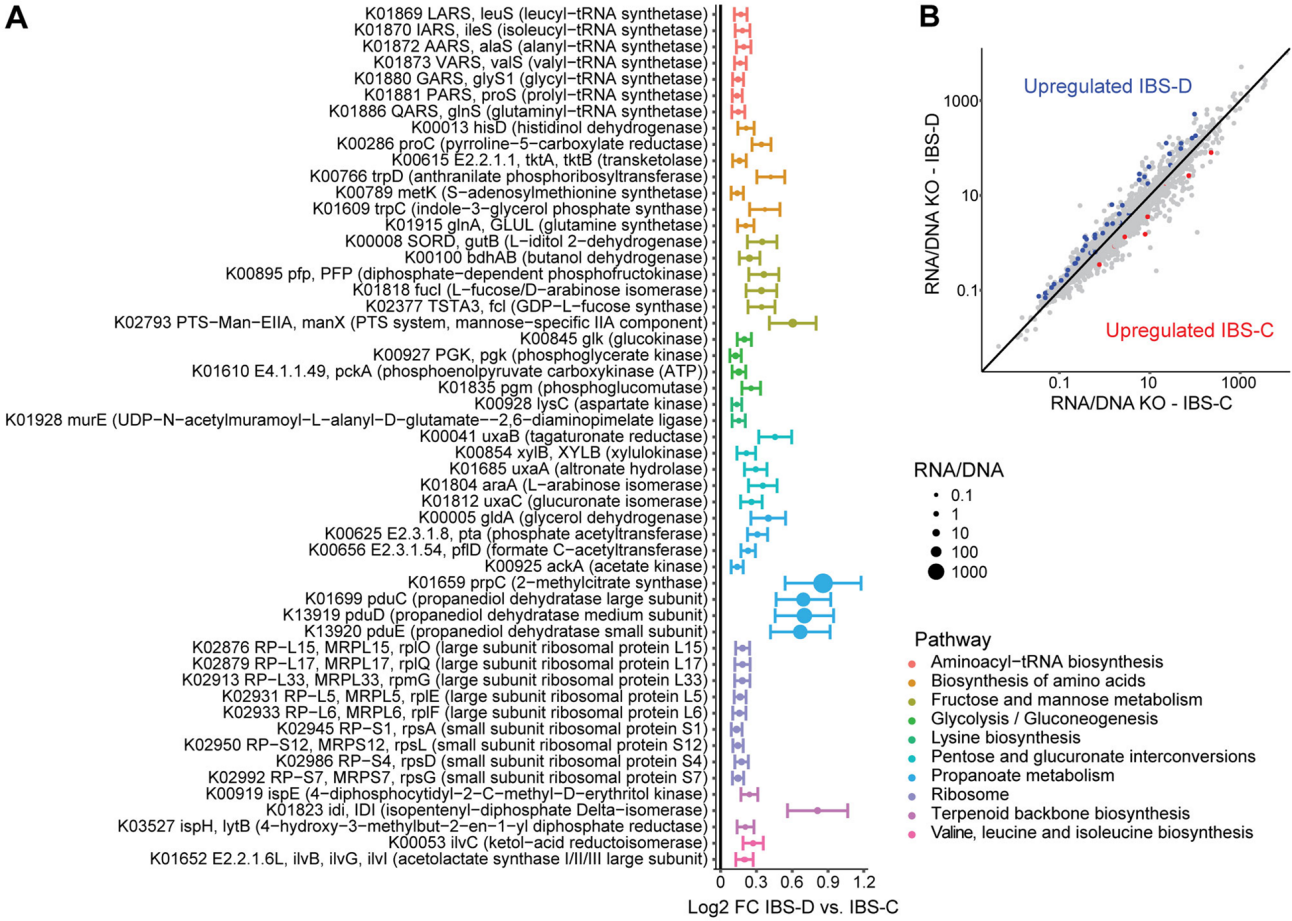
IBS-D is characterized by transcriptional upregulation relative to IBS-C of genes involved in proponoate metabolism, terpenoid biosynthesis, fructose and mannose metabolism, and glycolysis. **A** Pathways that were significantly enriched in transcripts differentially regulated in IBS-D vs. IBS-C were identified. Significant transcripts within these pathways (*q* < 0.25 adjusting for batch, age, sex, race/ethnicity, BMI, dietary category, and HAD-A) are shown, with color denoting pathway and dot size proportional to RNA/DNA ratio. Bars indicate standard error of log2 fold change estimates. **B** Transcript/gene abundance ratios are plotted for the 205 IBS-D and IBS-C subjects with both data types available. Transcripts that were significantly upregulated or downregulated in IBS-D compared to IBS-C are colored

Inter-omic analysis was then performed of gene-normalized transcript abundances to assess transcriptional regulation patterns in IBS-D vs. IBS-C. IBS-D was associated with significant shifts in 140 transcript RNA/DNA ratios, of which 128 were upregulated in IBS-D (Fig. 6, Additional file 1: Table S3). Pathway analysis demonstrated 10 pathways enriched for differentially abundant transcripts, including fructose and mannose metabolism, propanoate metabolism, aminoacyl-tRNA biosynthesis, terpenoid biosynthesis, and pentose and glucuronate interconversions. The most significantly upregulated transcripts were involved in proprionate metabolism, including 2-methylcitrate synthase, three subunits of propanediol dehydratase, and glycerol dehydrogenase. Upregulated fructose and mannose metabolism-related transcripts included those that had been enriched in the metatranscriptome without consideration of gene abundance (mannose phosphotransferases, L-iditol 2-dehydrogenase, L-fucose/D-arabinose isomerase) as well as others including diphosphate-dependent phosphofructokinase, GDP-L-fucose synthase, and butanol dehydrogenase.

Consistent with the functional shifts in the microbiome in IBS-D vs. IBS-C, there were 46 differentially abundant metabolites (Additional file 1: Figure S3C). This included increased levels of two bile acids (cholate and isoursodeoxycholate), polyamine products of amino acid fermentation (cadaverine, N-acetyl-cadaverine, N-acetylputrescine), carbohydrates (mannose, glucose, ribulose, glucuronate, ribose), intermediates in the citric acid cycle (malate, fumarate), and nucleotides (hypoxanthine, thymidine, 2'-deoxycytidine). IBS-D had decreased levels of fatty acids (myristate, palmitate, stearate), food components (carotene diol, phytanate, digalacturonic acid), phenylacetate, and the bacterial metabolites P-cresol and enterolactone. Of these metabolites, fumarate and hypoxanthine were significantly associated with microbial metabolic potential by both the metatranscriptome and predicted metagenome; glucose and nicotinate were associated with the metatranscriptome; and cholate, cadaverine, glucuronate, thymidine, and phenylacetate were associated with the predicted metagenome (Additional file 1: Figure S2).

**Fig. 7:**
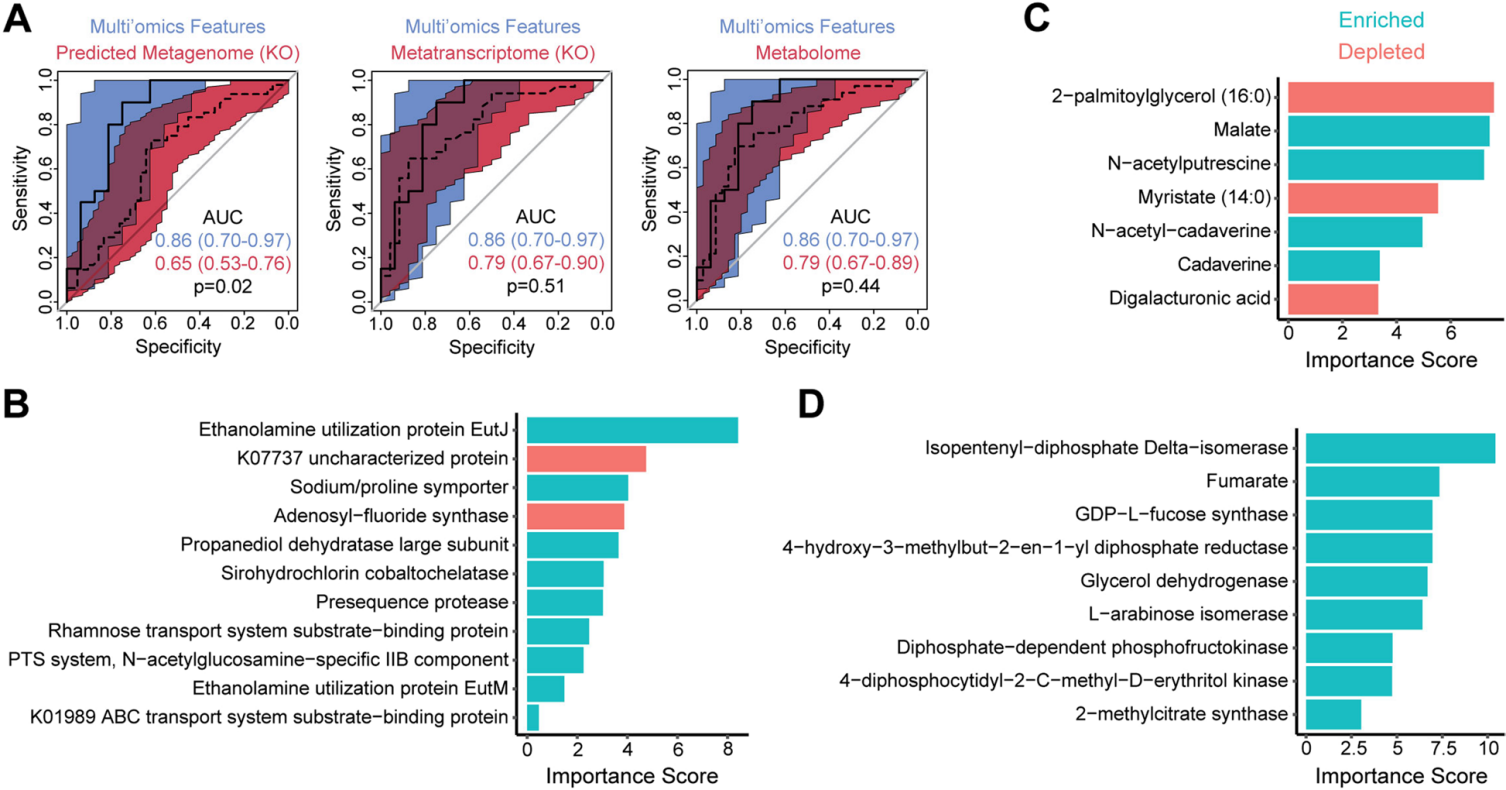
Metabolites, transcripts, and transcript/gene ratios can differentiate IBS-D from IBS-C with high accuracy. **A** ROC curves for random forest classifiers constructed from differentially abundant predicted genes, transcripts, and metabolites (colored in red) are compared to the ROC curve for a multi-omics classifier (colored in blue). The multi-omics classifier was constructed from significantly upregulated transcripts (by RNA/DNA ratio) in enriched pathways and differential metabolites associated with microbial community metabolic potential. All classifiers were trained on 60% of the dataset and tested on the remaining 40% of samples (*n* = 126 with all three data types). Colored areas indicate the 95% confidence intervals of the ROC curves. *P* values were calculated by bootstrapping. **B–D** Importance scores are shown for features included in the **B** metatranscriptomics, **C** metabolomics, and **D** multi-omics classifiers, with bar color denoting features that were enriched or depleted in IBS-D

We then assessed the ability of differential transcripts, metabolites, and multi-omics features (gene-normalized transcript abundances and differential metabolites with significant association with microbial metabolic potential) to differentiate IBS-D from IBS-C. The multi-omics classifier had the highest performance with AUC 0.86, which was significantly greater than that of the predicted metagenome, which only had AUC of 0.65 (Fig. 7A). Classifiers constructed from metatranscriptomics KOs and metabolites each had AUC 0.79, which was not significantly different from the multi-omics classifier. Random forests classifiers were not constructed using microbial composition (16S or metatranscriptomics taxonomy) given the lack of differentially abundant taxa. Transcripts in the metatranscriptomics classifier included ethanolamine utilization proteins, propanediol dehydratase, sodium/proline symporter, and N-acetylglucosamine phosphotransferase (Fig. 7B). The metabolomics classifier incorporated all three differential products of amino acid fermentation (cadaverine, N-acetyl-cadaverine, N-acetylputrescine), malate, fatty acids (mytristate and 2-palmitoylglycerol), and digalacturonic acid (a product of pectin breakdown) (Fig. 7C). The features contributing to the multi-omics classifier included fumarate and transcripts in pathways for fructose and mannose metabolism (diphosphate-dependent phosphofructokinase, GDP-L-fucose synthase), terpenoid biosynthesis (isopentenyl-diphosphate delta-isomerase, 4-hydroxy-3-methylbut-2-en-1-yl diphosphate reductase, 4-diphosphocytidyl-2-c-methyl-d-erythritol kinase), pentose and glucuronate interconversions (L-arabinose isomerase), and propionate metabolism (2-methylcitrate synthase, glycerol dehydrogenase) (Fig. 7D).

## Discussion

In this study, we present the results of the first large-scale metatranscriptomics analysis of a well-phenotyped IBS cohort and one of the two largest IBS cross-sectional global metabolomics studies to-date [15]. In combination with 16S rRNA sequencing data and predicted metagenomics, five datasets were analyzed to provide a comprehensive multi-omics assessment of microbial composition and function. Each dataset included microbial features that were significantly associated with IBS status but could only achieve modest accuracy in classification algorithms. Using multi-omics features that incorporated information from two related datasets (e.g., metatranscriptome KOs and predicted KO gene abundances, metabolites, and metatranscriptome metabolic potential), an accurate classifier for IBS vs. HC could be achieved. This supports that IBS is characterized by a distinct microbial functional state across a large cohort representative of the racial/ethnic diversity in the USA.

Compositional shifts in the microbiome of IBS compared to HC were observed based on both 16S relative abundances and transcriptional representation. A consistent finding in both 16S and metatranscriptomics data was increased *Bacteroides dorei*, which has not been previously reported in IBS. Both datasets also showed increased levels of *Actinomyces* spp., which is consistent with a large shotgun metagenomics study describing enrichment of multiple *Actinomyces* spp. including *A. odontolyticus* as seen here [7]. Additional species level microbial shifts detected by 16S sequencing or metatranscriptomics that have been previously noted in large shotgun metagenomics studies include increased *Streptococcus* species (*S. parasanguinis*), *Eggerthella lenta*, and *Blautia hydrogenotrophica* as well as reduced *Faecalibacterium prausnitzii*, *Bacteroides thetaiotaomicron*, and *Bilophila wadsworthia* [11, 16, 23]. Other taxonomic shifts observed in this study have not been previously reported and many taxa that were reported in prior studies were not significant in our study. We also did not observe reduced alpha diversity in IBS, which has been reported in some prior studies but not others [6–9]. These differences could reflect multiple factors that differentiated our study including racial/ethnic composition, recruitment of subjects from the community rather than specialty clinics, and differences in analytical approach. Importantly, this study differed from many prior ones in using multivariate strategies to adjust for a wide range of covariates differing between IBS and HC that could confound analyses [7, 11, 13–16].

Among the functional microbial features associated with IBS, the largest contribution to the multi-omics IBS signature came from three microbiome-related fecal metabolites: gentisate, hydrocinnamate, and tyramine. None of these or the other differentially abundant metabolites in IBS vs. HC have been previously implicated in IBS, possibly due to differences in metabolomics pipelines across studies which affect the specific metabolites that are detected and validated [13–15]. Gentisate, the most strongly predictive metabolite, is an intermediate in bacterial aerobic metabolism of aromatic compounds including hydroxybenzoates [46]. It has been reported to be undetectable in the serum of germ-free mice and strongly induced by bacterial colonization [47]. Gentisate inhibits fibroblast growth factor signaling to reduce tumor growth and associated angiogenesis [48]. It is unclear whether the decreased levels in IBS have any functional consequences for intestinal fibroblast growth factor signaling, which plays important roles in epithelial homeostasis and bile acid regulation [49]. Hydrocinnamate (3-phenylpropionate) is produced by microbial metabolism of polyphenols and serum levels of hydrocinnamate are positively associated with microbial alpha diversity [50–52]. IBS feces also showed reduced levels of benzoate and another member of the benzoate class, 3-(2-hydroxyphenol)propionate, which can also be derived from bacterial metabolism of polyphenols [53, 54]. These changes may reflect altered microbial metabolic activity or reduced polyphenol intake by IBS patients that was not captured by assessment of global dietary patterns. Tyramine is a biogenic amine that signals through trace amine-associated receptors. Bacteria can synthesize tyramine from tyrosine, and fecal tyramine levels are greatly increased by microbial colonization of germ-free mice and modulated by diet [55–57]. Tyramine induces intestinal contraction, mesenteric vasodilation, serotonin release by enterochromaffin cells, and inflammatory response by intestinal epithelial cells and macrophages, potential mechanisms by which it may promote symptoms in IBS [58–60]. Among the other differentially abundant metabolites, IBS was notably characterized by increased levels of many lipids including the bile acid glycodeoxycholate—which was previously reported to be elevated in IBS—and multiple free fatty acids [61]. Interestingly, IBS-D showed reduced free fatty acids compared to IBS-C. The increased levels of free fatty acids particularly in IBS-C may reflect increased delivery to the colon from dietary intake, altered intestinal epithelial lipid metabolism, or bacterial fatty acid metabolism. Gut microbes are thought to be unable to utilize fatty acids for energy harvest but are capable of synthesizing free fatty acids and using exogenous fatty acids for phospholipid biosynthesis [62, 63].

Despite the many metabolite associations with IBS and the strong contribution of three metabolites to the multi-omics classifier, the metabolomics only classifier had a modest AUC of 0.7 similar to what was reported in the two largest previously published fecal metabolomics studies [15, 16]. This supports that metabolic end product measurements need to be considered in the context of bacterial transcriptional profiles to accurately characterize the microbial metabolic state in IBS. We found that IBS was characterized by upregulation of transcripts involved in bacterial uptake and breakdown of fructooligosaccharides, polyols, and glucans. We further identified upregulation of bacterial enzymes involved in the succinate pathway of carbohydrate fermentation, which culminates in the production of propionate [45]. This increased capacity for carbohydrate utilization by the IBS microbiota is consistent with a prior large shotgun metagenomics study reporting increased carbohydrate degradation and fermentation pathways in IBS [7]. Moreover, a recent shotgun metagenomics reported that carbohydrate-metabolism associated genes (CAZy) were associated with symptom severity in IBS, further supporting a role for bacterial carbohydrate metabolism in IBS pathophysiology [64]. Preferential utilization of the succinate pathway in IBS for carbohydrate fermentation is supported by a meta-analysis of fecal short chain fatty acids (SCFAs) in IBS demonstrating increased proportion of propionate relative to other SCFAs [65]. Taken together, our findings and those of others support that gut microbes in IBS patients have increased capacity to utilize certain fermentable carbohydrates. Reliance of IBS-associated bacteria on this energy source may explain the clinical association of fermentable carbohydrates with IBS symptoms and clinical improvement with the FODMAPS diet, which restricts dietary intake of fermentable oligosaccharides, disaccharides, monosaccharides, and polyols [66]. It is important to note that there was low prevalence of FODMAPS diet in our IBS cohort (2%) and that IBS populations with greater prevalence of FODMAPS diet may not show the same transcriptional profile.

In addition to identifying metabolic features of IBS, we further found that IBS BH subtypes were robustly distinguished by functional microbial profiles. IBS-D was characterized by increased cholate and isoursodeoxycholate, which is consistent with prior studies reporting increased levels of cholate in IBS-D compared to IBS-C and bile acid malabsorption in IBS-D [14, 16]. Bile acids are known to induce diarrhea by triggering colonic chloride secretion, supporting direct involvement in the bowel habit phenotype of IBS-D [67]. Besides these bile acid shifts, IBS-D was characterized by upregulation of multiple metabolic pathways for bacterial energy harvest including transcripts involved in fructose, mannose, and polyol metabolism. This increased machinery for carbohydrate utilization corresponded to increased levels of multiple monosaccharides including mannose, glucose, ribulose, and ribose. IBS-D also had increased levels of intermediates of the succinate pathway of carbohydrate fermentation including fumarate and malate (succinate showed a non-significant increase and other intermediates were not detected), suggesting increased flux through this pathway compared to IBS-C. IBS-D also showed transcriptional upregulation of components of an alternative fermentation pathway to generate propionate via the 1,2-propanediol pathway. This included upregulation of three components of propanediol dehydratase as well as glycerol dehydrogenase, which participates in the generation of propanediol from monosaccharides [45]. Beyond carbohydrate metabolism, IBS-D also demonstrated evidence of increased amino acid catabolism based upon higher levels of several polyamines including cadaverine, N-acetylcadaverine, and N-acetylputrescine. Cadaverine and putrescine are products of arginine and lysine fermentation, respectively, which can further undergo N-acylation by gut microbes [68, 69]. Our findings are consistent with a prior study reporting elevated putrescine in IBS-D compared to IBS-C [13]. In addition, IBS-D demonstrated increased levels of three ethanolamine utilization proteins. Ethanolamine is released from breakdown of the membrane phospholipid phosphatidylethanolamine and has been reported to be metabolized primarily by enteric bacteria with pathogenic potential [70]. Overall, gut microbes in IBS-D demonstrate a profile consistent with increased utilization of multiple energy sources. It is unclear if these metabolic shifts promote symptoms in IBS-D or reflect adaptation of gut microbes to altered GI motility and secretion in IBS-D [71]. Of note, metabolites reported in other studies to differentiate IBS bowel habit subtypes such as tryptamine were not differentially abundant in this study, potentially reflecting the previously noted differences in study population and analytic strategy [14].

The strengths of this study include the large cohort size, inclusion of diverse racial/ethnic groups, extensive clinical phenotyping of IBS and control subjects, adjustment for relevant covariates in all analyses, and integration for the first time of metatranscriptomics and metabolomics to assess functional profiles in IBS. However, we acknowledge some limitations. Controls differed from IBS subjects in important parameters including sex, BMI, race/ethnicity, and diet. While considerable effort was made to account for these covariates, we cannot exclude residual effects of confounding. This study had a cross-sectional design, which may provide a less accurate assessment of complex microbial compositional and functional relationships compared to longitudinal studies [14]. Diet was assessed by a dietary pattern questionnaire, but this may not capture nutrient differences such as in polyphenol intake that may underlie observed microbial profiles. In addition, bacterial gene content was predicted from 16S rRNA gene sequencing rather than directly assessed by shotgun metagenomics. While predicted abundances of individual genes were not used directly as differential features for IBS or its BH subtypes, they were incorporated into gene-normalized transcriptional analyses. Predictions made using PICRUSt2 have been reported to have Spearman correlation of between 0.79 and 0.88 with shotgun metagenomics data, supporting that they are robust overall [25]. However, given the potential inaccuracies of gene abundance prediction, further studies incorporating shotgun metagenomics are required to confirm the findings of gene-normalized transcriptional analyses. Finally, the global metabolomics pipeline used for this study does not detect SCFAs, so we were unable to assess whether transcript patterns were associated with altered fecal levels of SCFAs.

## Conclusions

IBS is characterized by both compositional changes and robust shifts in microbial function that emerge from multi-omics integration of bacterial transcriptional patterns and fecal metabolite levels. The metabolic signature of IBS supports the utilization of multiple fermentable carbohydrates through the succinate pathway. This is consistent with the efficacy of interventions such as FODMAPS diet that exclude such energy sources. Additional novel metabolic pathways and metabolites such as tyramine were also identified. Further investigation of these pathways could elucidate mechanisms by which gut microbes contribute to IBS pathophysiology and inform our understanding of current microbiome-directed therapeutic strategies for IBS. Moreover, IBS BH subtypes showed strong functional differentiation, implying a critical role for microbial metabolic activity in dictating the bowel habit alterations seen in IBS. Our findings support the need for integrative assessment of microbial function incorporating both metabolomics and metatranscriptomics in IBS microbiome studies to identify relevant pathways that impact symptom generation and could represent novel mechanistic targets for microbiome-directed interventions.

## Supplementary Information

**Additional file 1: Figure S1.** Significant associations were seen across all pairwise combinations of datasets. (A) Procrustes analysis was used to superimpose dbRDA ordinations of the indicated pairs of datasets. IBS and HC samples are denoted by color. (B) Mantel test of association for all pairwise combinations of datasets. All were significant, with color indicating level of significance. **Figure S2.** Metabolites that were differentially abundant in IBS vs. HC or IBS-D vs. IBS-C and were associated with the gut microbiome by metabolic modeling. (A) Metabolites that were associated with the community metabolic potential (CMP) scores derived from predicted bacterial gene content are shown. Metabolomics and predicted metagenomics data were available for 361 subjects. Each sample is plotted by its CMP score and the log2 of the normalized metabolite level. The dashed lines represent linear regression of metabolite levels with CMP scores and the number in the upper right of each plot indicates the *R*^2^. (B) Metabolites that were associated with CMP scores derived from the metatranscriptome. Metabolomics and metatranscriptomics data were available for 234 subjects. **Figure S3.** IBS-D is differentiated from IBS-C by diverse functional shifts including increased polyamines, bile acids, glutamate synthesis, and ethanolamine utilization. (A) Differentially abundant taxa (*q*<0.25) in 16S sequencing (*n*=312) and metatranscriptomics (*n*=208) datasets adjusting for batch, age, sex, race/ethnicity, BMI, dietary category, and HAD-A. Effect size is shown as the log2 fold change (FC) of IBS-D compared to IBS-C. Dot size is proportional to abundance and color represents phylum. Bars indicate standard error of log2 fold change estimates. (B) Differentially abundant transcripts in IBS-D vs. IBS-C, colored by pathway with dot size proportional to abundance. (C) Metabolites that significantly differed in IBS-D vs. IBS-C by global metabolomics (*n*=229), colored by functional category. **Table S1.** Dietary patterns. **Table S2.** Differentially regulated transcripts in IBS vs. HC. **Table S3.** Differentially regulated transcripts in IBS-D vs. IBS-C.

**Additional file 2.**

**Additional file 3.**

**Additional file 4.**

## Data Availability

The 16S rRNA gene sequencing and metatranscriptomics data supporting the conclusions of this article are available in the NCBI Bioproject repository, PRJNA812699 (https://www.ncbi.nlm.nih.gov/bioproject/812699). The untargeted metabolomics data are included with this article as a supplementary data file.
