# Supplementary material for: Multi-omics profiles of the intestinal microbiome in irritable bowel syndrome and its bowel habit subtypes
Source: Microbiome. 2023 Jan 10;11:5. doi: 10.1186/s40168-022-01450-5 (PMC9830758; doi:10.1186/s40168-022-01450-5)
Supplement: Supplementary file 2 — Additional file 1: Figure S1. Significant associations were seen across all pairwise combinations of datasets. (A) Procrustes analysis was used to superimpose dbRDA ordinations of the indicated pairs of datasets. IBS and HC samples are denoted by color. (B) Mantel test of association for all pairwise combinations of datasets. All were significant, with color indicating level of significance. Figure S2. Metabolites that were differentially abundant in IBS vs. HC or IBS-D vs. IBS-C and were associated with the gut microbiome by metabolic modeling. (A) Metabolites that were associated with the community metabolic potential (CMP) scores derived from predicted bacterial gene content are shown. Metabolomics and predicted metagenomics data were available for 361 subjects. Each sample is plotted by its CMP score and the log2 of the normalized metabolite level. The dashed lines represent linear regression of metabolite levels with CMP scores and the number in the upper right of each plot indicates the R2. (B) Metabolites that were associated with CMP scores derived from the metatranscriptome. Metabolomics and metatranscriptomics data were available for 234 subjects. Figure S3. IBS-D is differentiated from IBS-C by diverse functional shifts including increased polyamines, bile acids, glutamate synthesis, and ethanolamine utilization. (A) Differentially abundant taxa (q<0.25) in 16S sequencing (n=312) and metatranscriptomics (n=208) datasets adjusting for batch, age, sex, race/ethnicity, BMI, dietary category, and HAD-A. Effect size is shown as the log2 fold change (FC) of IBS-D compared to IBS-C. Dot size is proportional to abundance and color represents phylum. Bars indicate standard error of log2 fold change estimates. (B) Differentially abundant transcripts in IBS-D vs. IBS-C, colored by pathway with dot size proportional to abundance. (C) Metabolites that significantly differed in IBS-D vs. IBS-C by global metabolomics (n=229), colored by functional category. Table S1. Di [file 40168_2022_1450_MOESM1_ESM.docx]

**SUPPLEMENTARY FIGURES AND TABLES**

**
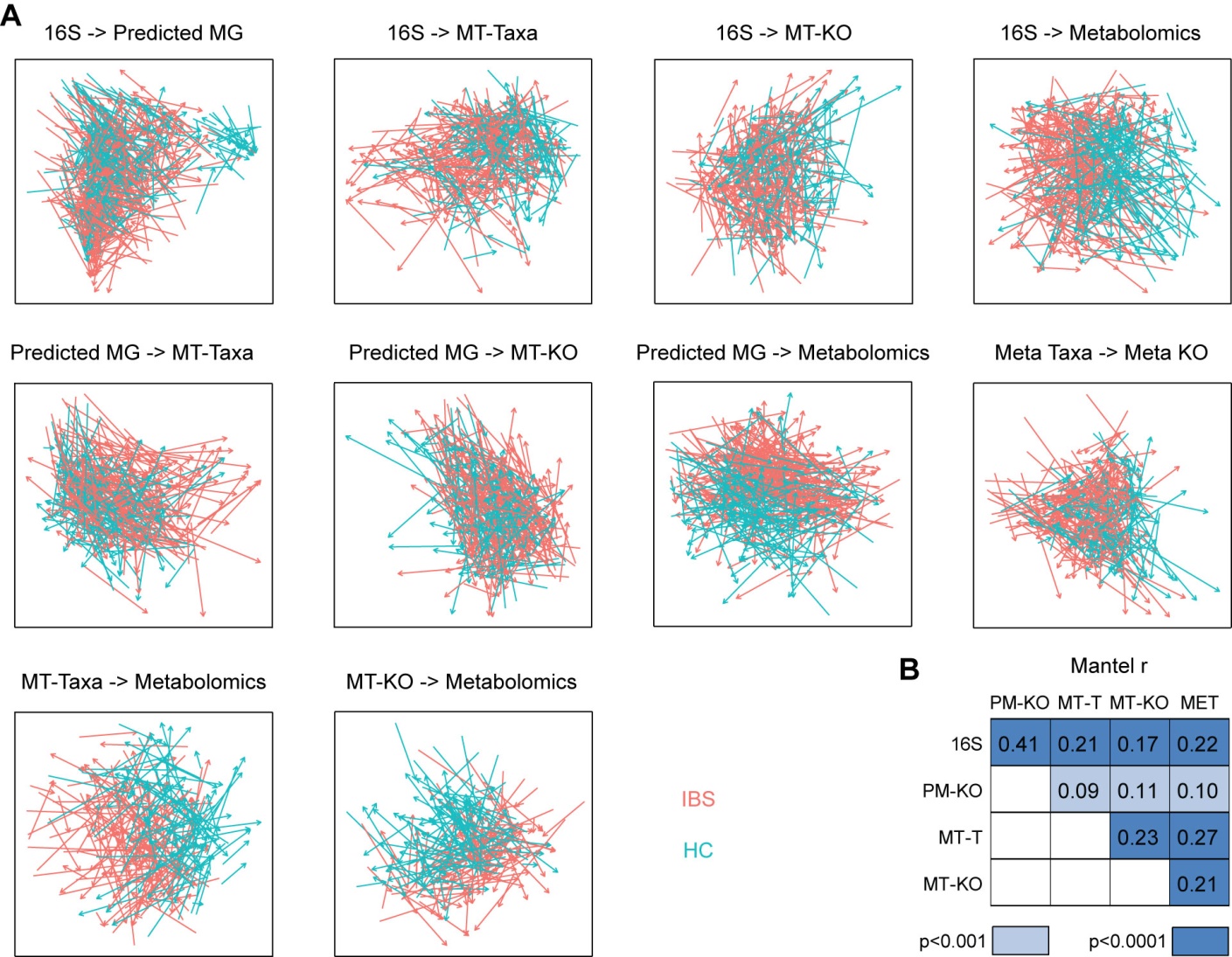
**

**Figure S1. Significant associations were seen across all pairwise combinations of datasets.** (**A**) Procrustes analysis was used to superimpose dbRDA ordinations of the indicated pairs of datasets. IBS and HC samples are denoted by color. (**B**) Mantel test of association for all pairwise combinations of datasets. All were significant, with color indicating level of significance.

**
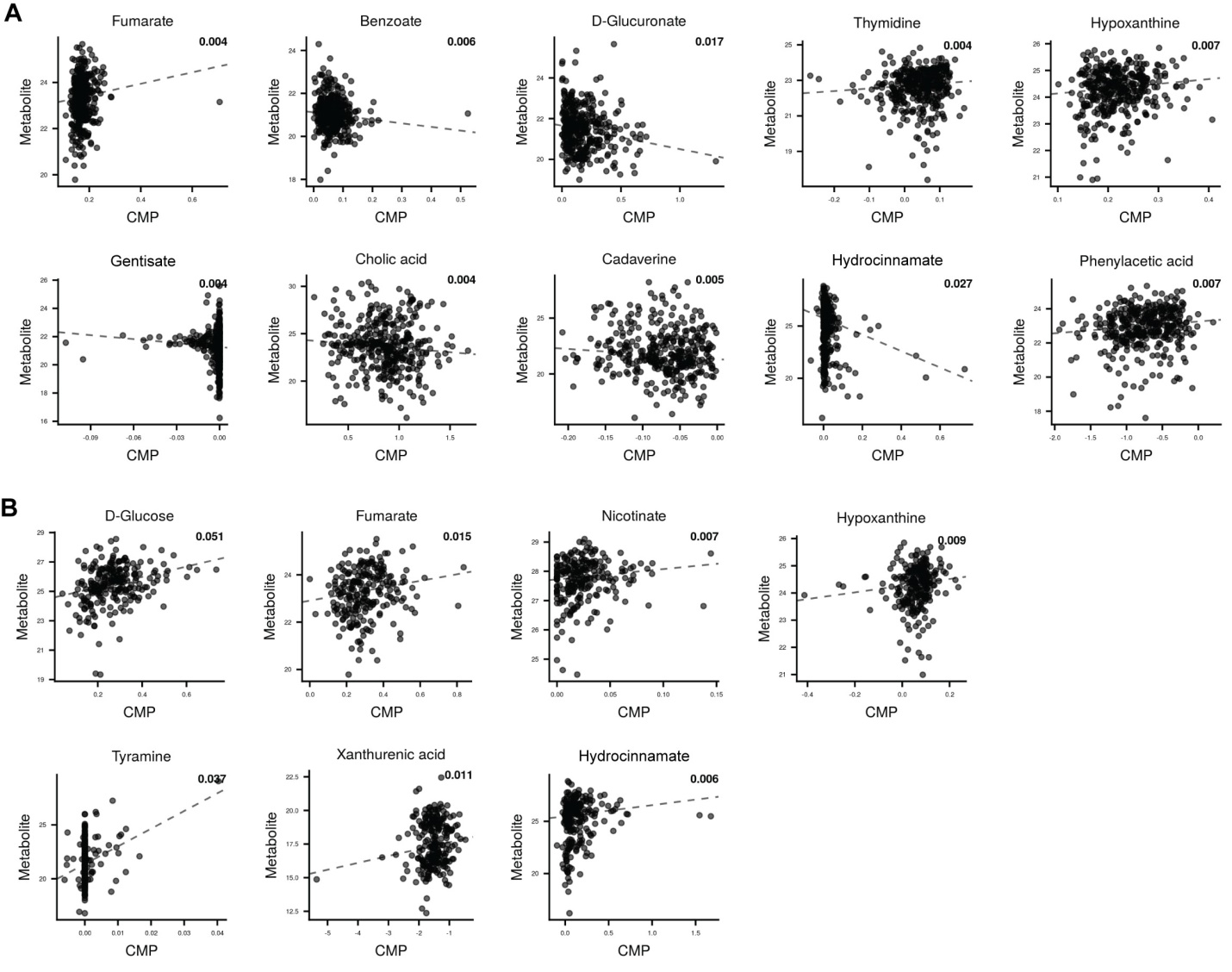
**

**Figure S2. Metabolites that were differentially abundant in IBS vs. HC or IBS-D vs. IBS-C and were associated with the gut microbiome by metabolic modeling.** (**A**) Metabolites that were associated with the community metabolic potential (CMP) scores derived from predicted bacterial gene content are shown. Metabolomics and predicted metagenomics data were available for 361 subjects. Each sample is plotted by its CMP score and the log2 of the normalized metabolite level. The dashed lines represent linear regression of metabolite levels with CMP scores and the number in the upper right of each plot indicates the R^2^. (**B**) Metabolites that were associated with CMP scores derived from the metatranscriptome. Metabolomics and metatranscriptomics data were available for 234 subjects.

**
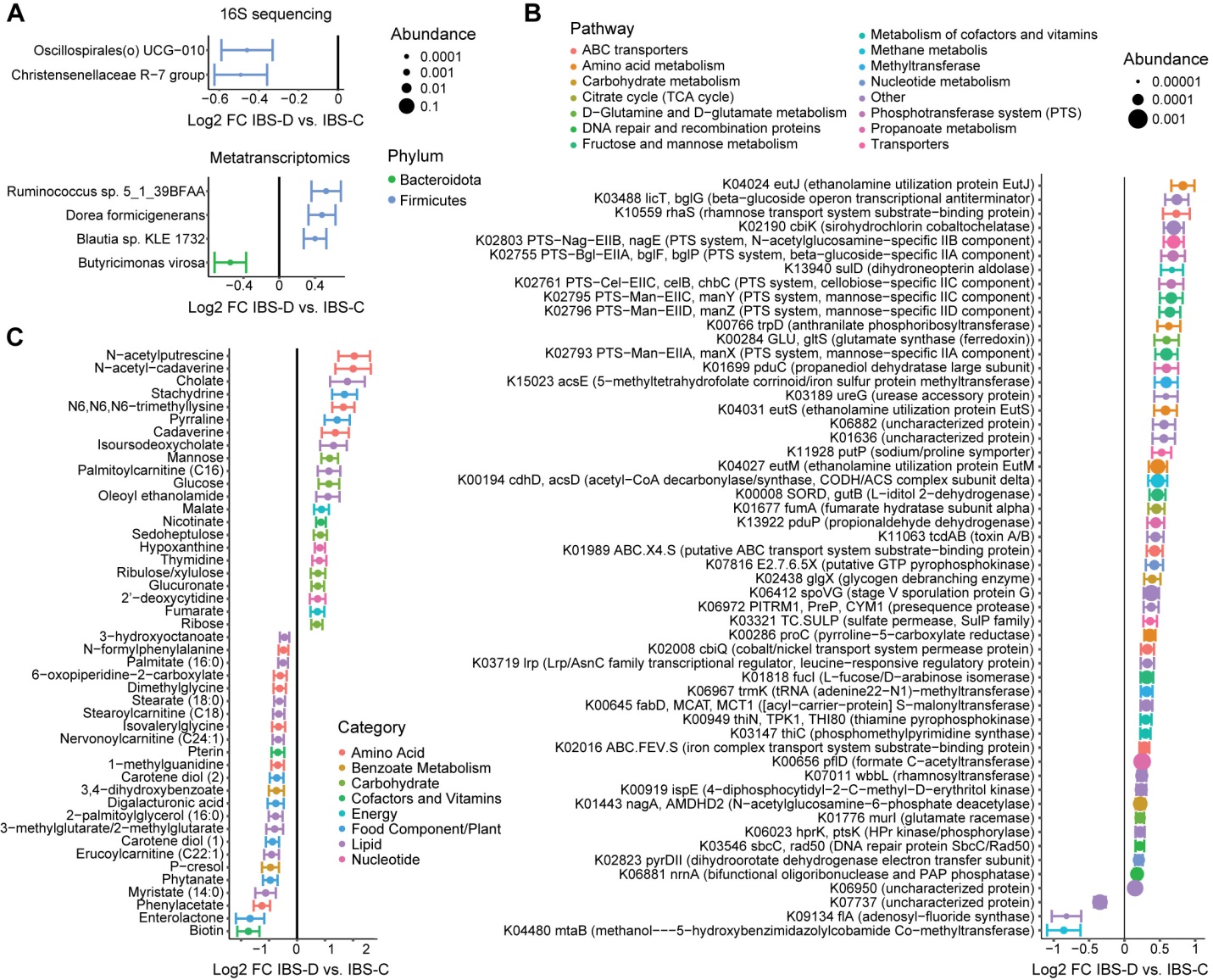
**

**Figure S3. IBS-D is differentiated from IBS-C by diverse functional shifts including increased polyamines, bile acids, glutamate synthesis, and ethanolamine utilization.** (**A**) Differentially abundant taxa (q<0.25) in 16S sequencing (n=312) and metatranscriptomics (n=208) datasets adjusting for batch, age, sex, race/ethnicity, BMI, dietary category, and HAD-A. Effect size is shown as the log2 fold change (FC) of IBS-D compared to IBS-C. Dot size is proportional to abundance and color represents phylum. Bars indicate standard error of log2 fold change estimates. (**B**) Differentially abundant transcripts in IBS-D vs. IBS-C, colored by pathway with dot size proportional to abundance. (**C**) Metabolites that significantly differed in IBS-D vs. IBS-C by global metabolomics (n=229), colored by functional category.

| **Table S1. Dietary patterns** | | | |
| --- | --- | --- | --- |
|  | **HC** | **IBS** | **P-value** |
| Standard |  |  |  |
| Standard American | 24% | 15% | **0.02** |
| Modified American | 44% | 33% | **0.03** |
| Mediterranean | 11% | 7% | 0.21 |
| Restrictive |  |  |  |
| Gluten-free | 3% | 15% | **0.0002** |
| Lactose-free | 4% | 13% | **0.003** |
| FODMAPS | 1% | 2% | 0.43 |
| Other |  |  |  |
| Paleo | 1% | 3% | 0.51 |
| Pescetarian | 4% | 4% | 1.0 |
| Vegetarian | 7% | 5% | 0.37 |
| Vegan | 1% | 2% | 1.0 |

| **Table S2. Differentially regulated transcripts in IBS vs. HC** | | | | |
| --- | --- | --- | --- | --- |
| **KEGG ID** | **Gene Symbol** | **Description** | **Log2 FC** | **Qvalue** |
| K03787 | surE | 5'-nucleotidase [EC:3.1.3.5] | 0.32 | 0.0004 |
| K00605 | gcvT, AMT | aminomethyltransferase [EC:2.1.2.10] | 0.29 | 0.001 |
| K00239 | sdhA, frdA | succinate dehydrogenase / fumarate reductase, flavoprotein subunit [EC:1.3.5.1 1.3.5.4] | 0.23 | 0.002 |
| K00347 | nqrB | Na+-transporting NADH:ubiquinone oxidoreductase subunit B [EC:7.2.1.1] | 0.37 | 0.003 |
| K01847 | MUT | methylmalonyl-CoA mutase [EC:5.4.99.2] | 0.29 | 0.003 |
| K03771 | surA | peptidyl-prolyl cis-trans isomerase SurA [EC:5.2.1.8] | 0.27 | 0.004 |
| K00428 | E1.11.1.5 | cytochrome c peroxidase [EC:1.11.1.5] | 0.46 | 0.004 |
| K00929 | buk | butyrate kinase [EC:2.7.2.7] | 0.29 | 0.004 |
| K00895 | pfp, PFP | diphosphate-dependent phosphofructokinase [EC:2.7.1.90] | 0.33 | 0.005 |
| K01186 | NEU1 | sialidase-1 [EC:3.2.1.18] | 0.30 | 0.006 |
| K08301 | rng, cafA | ribonuclease G [EC:3.1.26.-] | 0.18 | 0.008 |
| K03770 | ppiD | peptidyl-prolyl cis-trans isomerase D [EC:5.2.1.8] | 0.25 | 0.010 |
| K02440 | GLPF | glycerol uptake facilitator protein | -0.43 | 0.01 |
| K02437 | gcvH, GCSH | glycine cleavage system H protein | 0.24 | 0.01 |
| K06158 | ABCF3 | ATP-binding cassette, subfamily F, member 3 | 0.13 | 0.01 |
| K00677 | lpxA | UDP-N-acetylglucosamine acyltransferase [EC:2.3.1.129] | 0.25 | 0.02 |
| K03561 | exbB | biopolymer transport protein ExbB | 0.23 | 0.02 |
| K03281 | TC.CIC | chloride channel protein, CIC family | 0.24 | 0.02 |
| K03775 | slyD | FKBP-type peptidyl-prolyl cis-trans isomerase SlyD [EC:5.2.1.8] | 0.25 | 0.02 |
| K02484 | K02484 | two-component system, OmpR family, sensor kinase [EC:2.7.13.3] | 0.25 | 0.02 |
| K00180 | iorB | indolepyruvate ferredoxin oxidoreductase, beta subunit [EC:1.2.7.8] | 0.23 | 0.02 |
| K00179 | iorA | indolepyruvate ferredoxin oxidoreductase, alpha subunit [EC:1.2.7.8] | 0.20 | 0.03 |
| K03585 | acrA, mexA, adeI, smeD | membrane fusion protein, multidrug efflux system | 0.22 | 0.03 |
| K03773 | fklB | FKBP-type peptidyl-prolyl cis-trans isomerase FklB [EC:5.2.1.8] | 0.27 | 0.03 |
| K00241 | sdhC, frdC | succinate dehydrogenase / fumarate reductase, cytochrome b subunit | 0.29 | 0.03 |
| K06392 | spoIIIAC | stage III sporulation protein AC | -0.58 | 0.03 |
| K03313 | nhaA | Na+:H+ antiporter, NhaA family | 0.25 | 0.03 |
| K07164 | K07164 | uncharacterized protein | 0.20 | 0.04 |
| K01610 | E4.1.1.49, pckA | phosphoenolpyruvate carboxykinase (ATP) [EC:4.1.1.49] | 0.15 | 0.04 |
| K00350 | nqrE | Na+-transporting NADH:ubiquinone oxidoreductase subunit E [EC:7.2.1.1] | 0.32 | 0.04 |
| K00803 | AGPS, agpS | alkyldihydroxyacetonephosphate synthase [EC:2.5.1.26] | -0.56 | 0.04 |
| K03924 | moxR | MoxR-like ATPase [EC:3.6.3.-] | 0.15 | 0.05 |
| K07114 | yfbK | Ca-activated chloride channel homolog | 0.20 | 0.05 |
| K00634 | ptb | phosphate butyryltransferase [EC:2.3.1.19] | 0.23 | 0.05 |
| K00177 | korC, oorC | 2-oxoglutarate ferredoxin oxidoreductase subunit gamma [EC:1.2.7.3] | 0.20 | 0.05 |
| K02622 | parE | topoisomerase IV subunit B [EC:5.6.2.2] | 0.19 | 0.05 |
| K01745 | hutH, HAL | histidine ammonia-lyase [EC:4.3.1.3] | 0.24 | 0.05 |
| K00175 | korB, oorB, oforB | 2-oxoglutarate/2-oxoacid ferredoxin oxidoreductase subunit beta [EC:1.2.7.3 1.2.7.11] | 0.18 | 0.05 |
| K07588 | argK | LAO/AO transport system kinase [EC:2.7.-.-] | 0.21 | 0.05 |
| K02120 | ATPVD, ntpD, atpD | V/A-type H+/Na+-transporting ATPase subunit D | 0.14 | 0.05 |
| K09685 | purR | purine operon repressor | -0.55 | 0.06 |
| K01960 | pycB | pyruvate carboxylase subunit B [EC:6.4.1.1] | 0.21 | 0.06 |
| K00864 | glpK, GK | glycerol kinase [EC:2.7.1.30] | -0.18 | 0.06 |
| K02065 | mlaF, linL, mkl | phospholipid/cholesterol/gamma-HCH transport system ATP-binding protein | 0.22 | 0.06 |
| K03530 | hupB | DNA-binding protein HU-beta | 0.13 | 0.07 |
| K03269 | lpxH | UDP-2,3-diacylglucosamine hydrolase [EC:3.6.1.54] | 0.24 | 0.07 |
| K01840 | manB | phosphomannomutase [EC:5.4.2.8] | 0.19 | 0.07 |
| K01886 | QARS, glnS | glutaminyl-tRNA synthetase [EC:6.1.1.18] | 0.11 | 0.07 |
| K01284 | dcp | peptidyl-dipeptidase Dcp [EC:3.4.15.5] | 0.22 | 0.07 |
| K00940 | ndk, NME | nucleoside-diphosphate kinase [EC:2.7.4.6] | 0.24 | 0.07 |
| K09181 | yfiQ | acetyltransferase | 0.21 | 0.07 |
| K07277 | SAM50, TOB55, bamA | outer membrane protein insertion porin family | 0.20 | 0.07 |
| K01895 | ACSS, acs | acetyl-CoA synthetase [EC:6.2.1.1] | 0.19 | 0.07 |
| K00024 | mdh | malate dehydrogenase [EC:1.1.1.37] | 0.22 | 0.07 |
| K07713 | zraR, hydG | two-component system, NtrC family, response regulator HydG | 0.23 | 0.08 |
| K01155 | E3.1.21.4 | type II restriction enzyme [EC:3.1.21.4] | 0.26 | 0.08 |
| K01197 | hya | hyaluronoglucosaminidase [EC:3.2.1.35] | 0.26 | 0.08 |
| K00243 | K00243 | uncharacterized protein | 0.14 | 0.09 |
| K07306 | dmsA | anaerobic dimethyl sulfoxide reductase subunit A [EC:1.8.5.3] | 0.60 | 0.09 |
| K05801 | djlA | DnaJ like chaperone protein | 0.31 | 0.09 |
| K01966 | PCCB, pccB | propionyl-CoA carboxylase beta chain [EC:6.4.1.3 2.1.3.15] | 0.23 | 0.09 |
| K03701 | uvrA | excinuclease ABC subunit A | 0.09 | 0.09 |
| K00639 | kbl, GCAT | glycine C-acetyltransferase [EC:2.3.1.29] | 0.18 | 0.09 |
| K01897 | ACSL, fadD | long-chain acyl-CoA synthetase [EC:6.2.1.3] | 0.13 | 0.09 |
| K05985 | rnmV | ribonuclease M5 [EC:3.1.26.8] | -0.28 | 0.10 |
| K00348 | nqrC | Na+-transporting NADH:ubiquinone oxidoreductase subunit C [EC:7.2.1.1] | 0.26 | 0.10 |
| K00666 | ACSF2 | fatty-acyl-CoA synthase [EC:6.2.1.-] | 0.22 | 0.10 |
| K03801 | lipB | lipoyl(octanoyl) transferase [EC:2.3.1.181] | 0.25 | 0.10 |
| K04086 | clpL | ATP-dependent Clp protease ATP-binding subunit ClpL | -0.44 | 0.10 |
| K02527 | kdtA, waaA | 3-deoxy-D-manno-octulosonic-acid transferase [EC:2.4.99.12 2.4.99.13 2.4.99.14 2.4.99.15] | 0.21 | 0.11 |
| K01486 | ade | adenine deaminase [EC:3.5.4.2] | -0.20 | 0.11 |
| K00346 | nqrA | Na+-transporting NADH:ubiquinone oxidoreductase subunit A [EC:7.2.1.1] | 0.28 | 0.11 |
| K08965 | mtnW | 2,3-diketo-5-methylthiopentyl-1-phosphate enolase [EC:5.3.2.5] | -1.05 | 0.11 |
| K00174 | korA, oorA, oforA | 2-oxoglutarate/2-oxoacid ferredoxin oxidoreductase subunit alpha [EC:1.2.7.3 1.2.7.11] | 0.17 | 0.11 |
| K03296 | TC.HAE1 | hydrophobic/amphiphilic exporter-1 (mainly G- bacteria), HAE1 family | 0.16 | 0.11 |
| K07053 | E3.1.3.97 | 3',5'-nucleoside bisphosphate phosphatase [EC:3.1.3.97] | -0.13 | 0.11 |
| K00240 | sdhB, frdB | succinate dehydrogenase / fumarate reductase, iron-sulfur subunit [EC:1.3.5.1 1.3.5.4] | 0.18 | 0.11 |
| K01883 | CARS, cysS | cysteinyl-tRNA synthetase [EC:6.1.1.16] | 0.10 | 0.11 |
| K01190 | lacZ | beta-galactosidase [EC:3.2.1.23] | 0.14 | 0.12 |
| K00351 | nqrF | Na+-transporting NADH:ubiquinone oxidoreductase subunit F [EC:7.2.1.1] | 0.26 | 0.12 |
| K11085 | msbA | ATP-binding cassette, subfamily B, bacterial MsbA [EC:3.6.3.-] | 0.19 | 0.12 |
| K03693 | pbp1b | penicillin-binding protein 1B | -0.62 | 0.12 |
| K02355 | fusA, GFM, EFG | elongation factor G | 0.08 | 0.12 |
| K03534 | rhaM | L-rhamnose mutarotase [EC:5.1.3.32] | 0.28 | 0.12 |
| K00029 | maeB | malate dehydrogenase (oxaloacetate-decarboxylating)(NADP+) [EC:1.1.1.40] | 0.21 | 0.13 |
| K00748 | lpxB | lipid-A-disaccharide synthase [EC:2.4.1.182] | 0.21 | 0.13 |
| K06861 | lptB | lipopolysaccharide export system ATP-binding protein [EC:3.6.3.-] | 0.20 | 0.13 |
| K01278 | DPP4, CD26 | dipeptidyl-peptidase 4 [EC:3.4.14.5] | 0.18 | 0.13 |
| K07001 | K07001 | NTE family protein | 0.14 | 0.14 |
| K03559 | exbD | biopolymer transport protein ExbD | 0.20 | 0.14 |
| K00257 | mbtN, fadE14 | acyl-ACP dehydrogenase [EC:1.3.99.-] | 0.25 | 0.14 |
| K00425 | cydA | cytochrome bd ubiquinol oxidase subunit I [EC:7.1.1.7] | 0.21 | 0.14 |
| K06180 | rluD | 23S rRNA pseudouridine1911/1915/1917 synthase [EC:5.4.99.23] | 0.09 | 0.14 |
| K03742 | pncC | nicotinamide-nucleotide amidase [EC:3.5.1.42] | -0.15 | 0.14 |
| K11744 | tqsA | AI-2 transport protein TqsA | 1.05 | 0.15 |
| K02621 | parC | topoisomerase IV subunit A [EC:5.6.2.2] | 0.17 | 0.15 |
| K08221 | yitG, ymfD, yfmO | MFS transporter, ACDE family, multidrug resistance protein | -0.85 | 0.15 |
| K01676 | fumA, fumB | fumarate hydratase, class I [EC:4.2.1.2] | 0.19 | 0.15 |
| K07182 | K07182 | CBS domain-containing protein | -0.73 | 0.15 |
| K06385 | spoIIP | stage II sporulation protein P | -0.28 | 0.15 |
| K01270 | pepD | dipeptidase D [EC:3.4.13.-] | 0.11 | 0.15 |
| K03474 | pdxJ | pyridoxine 5-phosphate synthase [EC:2.6.99.2] | 0.22 | 0.15 |
| K03332 | fruA | fructan beta-fructosidase [EC:3.2.1.80] | 0.36 | 0.15 |
| K13626 | fliW | flagellar assembly factor FliW | -0.32 | 0.16 |
| K06012 | gpr | spore protease [EC:3.4.24.78] | -0.28 | 0.16 |
| K01560 | E3.8.1.2 | 2-haloacid dehalogenase [EC:3.8.1.2] | -0.26 | 0.16 |
| K06951 | K06951 | uncharacterized protein | -0.63 | 0.16 |
| K03832 | tonB | periplasmic protein TonB | 0.18 | 0.16 |
| K00339 | nuoJ | NADH-quinone oxidoreductase subunit J [EC:7.1.1.2] | 0.27 | 0.16 |
| K01639 | nanA, NPL | N-acetylneuraminate lyase [EC:4.1.3.3] | 0.17 | 0.16 |
| K02838 | frr, MRRF, RRF | ribosome recycling factor | 0.09 | 0.16 |
| K03648 | UNG, UDG | uracil-DNA glycosylase [EC:3.2.2.27] | 0.14 | 0.16 |
| K00812 | aspB | aspartate aminotransferase [EC:2.6.1.1] | 0.16 | 0.17 |
| K00219 | fadH | 2,4-dienoyl-CoA reductase (NADPH2) [EC:1.3.1.34] | 0.41 | 0.17 |
| K02508 | hpaA | AraC family transcriptional regulator, 4-hydroxyphenylacetate 3-monooxygenase operon regulatory protein | 0.54 | 0.17 |
| K01673 | cynT, can | carbonic anhydrase [EC:4.2.1.1] | 0.18 | 0.17 |
| K12373 | HEXA_B | hexosaminidase [EC:3.2.1.52] | 0.18 | 0.17 |
| K02029 | ABC.PA.P | polar amino acid transport system permease protein | -0.20 | 0.17 |
| K05832 | ABC.X4.P | putative ABC transport system permease protein | -0.25 | 0.17 |
| K05878 | dhaK | phosphoenolpyruvate---glycerone phosphotransferase subunit DhaK [EC:2.7.1.121] | -0.28 | 0.17 |
| K06905 | K06905 | uncharacterized protein | -0.54 | 0.17 |
| K03797 | prc, ctpA | carboxyl-terminal processing protease [EC:3.4.21.102] | 0.09 | 0.17 |
| K00831 | serC, PSAT1 | phosphoserine aminotransferase [EC:2.6.1.52] | 0.11 | 0.17 |
| K11618 | liaR | two-component system, NarL family, response regulator LiaR | -0.46 | 0.18 |
| K13747 | nspC | carboxynorspermidine decarboxylase [EC:4.1.1.96] | 0.16 | 0.18 |
| K12257 | secDF | SecD/SecF fusion protein | 0.14 | 0.18 |
| K03188 | ureF | urease accessory protein | -0.52 | 0.18 |
| K08218 | ampG | MFS transporter, PAT family, beta-lactamase induction signal transducer AmpG | 0.24 | 0.18 |
| K01187 | malZ | alpha-glucosidase [EC:3.2.1.20] | 0.15 | 0.18 |
| K02481 | K02481 | two-component system, NtrC family, response regulator | 0.21 | 0.18 |
| K07263 | pqqL | zinc protease [EC:3.4.24.-] | 0.19 | 0.18 |
| K07707 | agrA, blpR, fsrA | two-component system, LytTR family, response regulator AgrA | -0.52 | 0.18 |
| K01613 | psd, PISD | phosphatidylserine decarboxylase [EC:4.1.1.65] | 0.15 | 0.18 |
| K02199 | ccmG, dsbE | cytochrome c biogenesis protein CcmG, thiol:disulfide interchange protein DsbE | 0.42 | 0.19 |
| K00850 | pfkA, PFK | 6-phosphofructokinase 1 [EC:2.7.1.11] | 0.11 | 0.19 |
| K01997 | livH | branched-chain amino acid transport system permease protein | -0.22 | 0.19 |
| K00721 | DPM1 | dolichol-phosphate mannosyltransferase [EC:2.4.1.83] | 0.11 | 0.19 |
| K03572 | mutL | DNA mismatch repair protein MutL | 0.10 | 0.19 |
| K02523 | ispB | octaprenyl-diphosphate synthase [EC:2.5.1.90] | 0.19 | 0.19 |
| K07289 | asmA | AsmA protein | 0.44 | 0.19 |
| K01866 | YARS, tyrS | tyrosyl-tRNA synthetase [EC:6.1.1.1] | 0.09 | 0.20 |
| K00097 | pdxA | 4-hydroxythreonine-4-phosphate dehydrogenase [EC:1.1.1.262] | 0.16 | 0.20 |
| K09810 | lolD | lipoprotein-releasing system ATP-binding protein [EC:3.6.3.-] | 0.20 | 0.20 |
| K02837 | prfC | peptide chain release factor 3 | 0.11 | 0.20 |
| K00127 | fdoI, fdsG | formate dehydrogenase subunit gamma | 0.65 | 0.20 |
| K02357 | tsf, TSFM | elongation factor Ts | 0.08 | 0.21 |
| K01668 | E4.1.99.2 | tyrosine phenol-lyase [EC:4.1.99.2] | -0.76 | 0.21 |
| K11537 | xapB | MFS transporter, NHS family, xanthosine permease | 0.22 | 0.21 |
| K01858 | INO1, ISYNA1 | myo-inositol-1-phosphate synthase [EC:5.5.1.4] | 0.22 | 0.21 |
| K02564 | nagB, GNPDA | glucosamine-6-phosphate deaminase [EC:3.5.99.6] | 0.09 | 0.21 |
| K12264 | norV | anaerobic nitric oxide reductase flavorubredoxin | 0.49 | 0.21 |
| K00657 | speG, SAT | diamine N-acetyltransferase [EC:2.3.1.57] | 0.21 | 0.21 |
| K03654 | recQ | ATP-dependent DNA helicase RecQ [EC:3.6.4.12] | 0.11 | 0.21 |
| K05606 | MCEE, epi | methylmalonyl-CoA/ethylmalonyl-CoA epimerase [EC:5.1.99.1] | 0.21 | 0.22 |
| K09989 | K09989 | uncharacterized protein | -0.41 | 0.22 |
| K00355 | NQO1 | NAD(P)H dehydrogenase (quinone) [EC:1.6.5.2] | -0.59 | 0.22 |
| K00687 | pbp2B, penA | penicillin-binding protein 2B | -0.74 | 0.22 |
| K00956 | cysN | sulfate adenylyltransferase subunit 1 [EC:2.7.7.4] | 0.19 | 0.22 |
| K01770 | ispF | 2-C-methyl-D-erythritol 2,4-cyclodiphosphate synthase [EC:4.6.1.12] | 0.13 | 0.22 |
| K07386 | pepO | putative endopeptidase [EC:3.4.24.-] | 0.16 | 0.22 |
| K02687 | prmA | ribosomal protein L11 methyltransferase [EC:2.1.1.-] | 0.10 | 0.22 |
| K04078 | groES, HSPE1 | chaperonin GroES | -0.18 | 0.22 |
| K00176 | korD, oorD | 2-oxoglutarate ferredoxin oxidoreductase subunit delta [EC:1.2.7.3] | 0.20 | 0.23 |
| K01802 | E5.2.1.8 | peptidylprolyl isomerase [EC:5.2.1.8] | 0.20 | 0.23 |
| K03712 | marR | MarR family transcriptional regulator, multiple antibiotic resistance protein MarR | 0.46 | 0.23 |
| K13288 | orn, REX2, REXO2 | oligoribonuclease [EC:3.1.-.-] | -0.59 | 0.24 |
| K00281 | GLDC, gcvP | glycine dehydrogenase [EC:1.4.4.2] | 0.20 | 0.24 |
| K02429 | fucP | MFS transporter, FHS family, L-fucose permease | 0.18 | 0.24 |
| K08682 | acpH | acyl carrier protein phosphodiesterase [EC:3.1.4.14] | 0.68 | 0.24 |
| K12063 | traC | conjugal transfer ATP-binding protein TraC | 0.52 | 0.24 |
| K00970 | pcnB | poly(A) polymerase [EC:2.7.7.19] | 0.18 | 0.24 |
| K07278 | tamA | translocation and assembly module TamA | 0.39 | 0.24 |
| K03818 | wcaF | putative colanic acid biosynthesis acetyltransferase WcaF [EC:2.3.1.-] | -0.57 | 0.24 |
| K00951 | relA | GTP pyrophosphokinase [EC:2.7.6.5] | 0.07 | 0.24 |
| K01273 | DPEP | membrane dipeptidase [EC:3.4.13.19] | -0.18 | 0.24 |
| K01712 | hutU, UROC1 | urocanate hydratase [EC:4.2.1.49] | 0.18 | 0.24 |
| K00208 | fabI | enoyl-[acyl-carrier protein] reductase I [EC:1.3.1.9 1.3.1.10] | 0.19 | 0.24 |
| K00343 | nuoN | NADH-quinone oxidoreductase subunit N [EC:7.1.1.2] | 0.21 | 0.24 |
| K14652 | ribBA | 3,4-dihydroxy 2-butanone 4-phosphate synthase / GTP cyclohydrolase II [EC:4.1.99.12 3.5.4.25] | 0.12 | 0.24 |
| K03979 | obgE, cgtA, MTG2 | GTPase [EC:3.6.5.-] | 0.09 | 0.24 |
| K01492 | purNH | phosphoribosylglycinamide/phosphoribosylaminoimidazolecarboxamide formyltransferase [EC:2.1.2.2 2.1.2.3] | 0.20 | 0.25 |
| K08999 | K08999 | uncharacterized protein | 0.19 | 0.25 |

| **Table S3. Differentially regulated transcripts in IBS-D vs. IBS-C** | | | | |
| --- | --- | --- | --- | --- |
| **KEGG ID** | **Gene Symbol** | **Description** | **Log2FC** | **Qvalue** |
| K02227 | cbiB, cobD | adenosylcobinamide-phosphate synthase [EC:6.3.1.10] | 0.00 | 0.003 |
| K00286 | proC | pyrroline-5-carboxylate reductase [EC:1.5.1.2] | 0.00 | 0.004 |
| K06950 | K06950 | uncharacterized protein | 0.00 | 0.004 |
| K02190 | cbiK | sirohydrochlorin cobaltochelatase [EC:4.99.1.3] | 0.00 | 0.004 |
| K00949 | thiN, TPK1, THI80 | thiamine pyrophosphokinase [EC:2.7.6.2] | 0.00 | 0.004 |
| K06881 | nrnA | bifunctional oligoribonuclease and PAP phosphatase NrnA [EC:3.1.3.7 3.1.13.3] | 0.00 | 0.007 |
| K02823 | pyrDII | dihydroorotate dehydrogenase electron transfer subunit | 0.00 | 0.009 |
| K00656 | E2.3.1.54, pflD | formate C-acetyltransferase [EC:2.3.1.54] | 0.00 | 0.02 |
| K01776 | murI | glutamate racemase [EC:5.1.1.3] | 0.00 | 0.02 |
| K02016 | ABC.FEV.S | iron complex transport system substrate-binding protein | 0.00 | 0.02 |
| K03147 | thiC | phosphomethylpyrimidine synthase [EC:4.1.99.17] | 0.00 | 0.02 |
| K00645 | fabD, MCAT, MCT1 | [acyl-carrier-protein] S-malonyltransferase [EC:2.3.1.39] | 0.00 | 0.02 |
| K00625 | E2.3.1.8, pta | phosphate acetyltransferase [EC:2.3.1.8] | 0.00 | 0.03 |
| K07011 | wbbL | rhamnosyltransferase | 0.00 | 0.03 |
| K00766 | trpD | anthranilate phosphoribosyltransferase [EC:2.4.2.18] | 0.00 | 0.04 |
| K03817 | rimL | ribosomal-protein-serine acetyltransferase [EC:2.3.1.-] | 0.00 | 0.04 |
| K02199 | ccmG, dsbE | cytochrome c biogenesis protein CcmG, thiol:disulfide interchange protein DsbE | 0.00 | 0.05 |
| K00919 | ispE | 4-diphosphocytidyl-2-C-methyl-D-erythritol kinase [EC:2.7.1.148] | 0.00 | 0.05 |
| K04024 | eutJ | ethanolamine utilization protein EutJ | 0.00 | 0.05 |
| K01873 | VARS, valS | valyl-tRNA synthetase [EC:6.1.1.9] | 0.00 | 0.06 |
| K02950 | RP-S12, MRPS12 | small subunit ribosomal protein S12 | 0.00 | 0.06 |
| K00845 | glk | glucokinase [EC:2.7.1.2] | 0.00 | 0.06 |
| K00041 | uxaB | tagaturonate reductase [EC:1.1.1.58] | 0.00 | 0.06 |
| K01881 | PARS, proS | prolyl-tRNA synthetase [EC:6.1.1.15] | 0.00 | 0.07 |
| K00595 | cobL | precorrin-6Y C5,15-methyltransferase (decarboxylating) [EC:2.1.1.132] | 0.00 | 0.07 |
| K01006 | ppdK | pyruvate, orthophosphate dikinase [EC:2.7.9.1] | 0.00 | 0.07 |
| K02106 | atoE | short-chain fatty acids transporter | 0.00 | 0.07 |
| K02931 | RP-L5, MRPL5, rplE | large subunit ribosomal protein L5 | 0.00 | 0.07 |
| K01835 | pgm | phosphoglucomutase [EC:5.4.2.2] | 0.00 | 0.07 |
| K00928 | lysC | aspartate kinase [EC:2.7.2.4] | 0.00 | 0.07 |
| K09807 | K09807 | uncharacterized protein | 0.00 | 0.07 |
| K03521 | fixA, etfB | electron transfer flavoprotein beta subunit | 0.00 | 0.08 |
| K02048 | cysP | sulfate/thiosulfate transport system substrate-binding protein | 0.00 | 0.08 |
| K03522 | fixB, etfA | electron transfer flavoprotein alpha subunit | 0.00 | 0.08 |
| K04085 | tusA, sirA | tRNA 2-thiouridine synthesizing protein A [EC:2.8.1.-] | 0.00 | 0.08 |
| K00053 | ilvC | ketol-acid reductoisomerase [EC:1.1.1.86] | 0.00 | 0.08 |
| K11104 | melB | melibiose permease | 0.00 | 0.09 |
| K07027 | K07027 | glycosyltransferase 2 family protein | 0.00 | 0.09 |
| K01880 | GARS, glyS1 | glycyl-tRNA synthetase [EC:6.1.1.14] | 0.00 | 0.09 |
| K01869 | LARS, leuS | leucyl-tRNA synthetase [EC:6.1.1.4] | 0.00 | 0.09 |
| K01872 | AARS, alaS | alanyl-tRNA synthetase [EC:6.1.1.7] | 0.00 | 0.09 |
| K02986 | RP-S4, rpsD | small subunit ribosomal protein S4 | 0.00 | 0.10 |
| K00013 | hisD | histidinol dehydrogenase [EC:1.1.1.23] | 0.00 | 0.10 |
| K01823 | idi, IDI | isopentenyl-diphosphate Delta-isomerase [EC:5.3.3.2] | 0.00 | 0.10 |
| K02876 | RP-L15, MRPL15 | large subunit ribosomal protein L15 | 0.00 | 0.10 |
| K12990 | rfbF, rhlC | rhamnosyltransferase [EC:2.4.1.-] | 0.00 | 0.10 |
| K01685 | uxaA | altronate hydrolase [EC:4.2.1.7] | 0.00 | 0.10 |
| K03040 | rpoA | DNA-directed RNA polymerase subunit alpha [EC:2.7.7.6] | 0.00 | 0.10 |
| K02771 | PTS-Fru1-EIID, levG | PTS system, fructose-specific IID component | 0.00 | 0.10 |
| K07498 | K07498 | putative transposase | 0.00 | 0.10 |
| K03930 | estA | putative tributyrin esterase [EC:3.1.1.-] | 0.00 | 0.10 |
| K01915 | glnA, GLUL | glutamine synthetase [EC:6.3.1.2] | 0.00 | 0.11 |
| K02793 | PTS-Man-EIIA, manX | PTS system, mannose-specific IIA component [EC:2.7.1.191] | 0.00 | 0.11 |
| K02992 | RP-S7, MRPS7, rpsG | small subunit ribosomal protein S7 | 0.00 | 0.11 |
| K01740 | metY | O-acetylhomoserine (thiol)-lyase [EC:2.5.1.49] | 0.00 | 0.11 |
| K01955 | carB, CPA2 | carbamoyl-phosphate synthase large subunit [EC:6.3.5.5] | 0.00 | 0.11 |
| K02601 | nusG | transcription termination/antitermination protein NusG | 0.00 | 0.12 |
| K03925 | mraZ | MraZ protein | 0.00 | 0.12 |
| K02945 | RP-S1, rpsA | small subunit ribosomal protein S1 | 0.00 | 0.12 |
| K02377 | TSTA3, fcl | GDP-L-fucose synthase [EC:1.1.1.271] | 0.00 | 0.13 |
| K01699 | pduC | propanediol dehydratase large subunit [EC:4.2.1.28] | 0.00 | 0.13 |
| K11184 | chr, crh | catabolite repression HPr-like protein | 0.00 | 0.13 |
| K06518 | cidA | holin-like protein | 0.00 | 0.13 |
| K01925 | murD | UDP-N-acetylmuramoylalanine--D-glutamate ligase [EC:6.3.2.9] | 0.00 | 0.13 |
| K10843 | ERCC3, XPB | DNA excision repair protein ERCC-3 [EC:3.6.4.12] | 0.00 | 0.13 |
| K07502 | yprB | uncharacterized protein | 0.00 | 0.13 |
| K03439 | trmB, METTL1 | tRNA (guanine-N7-)-methyltransferase [EC:2.1.1.33] | 0.00 | 0.14 |
| K01870 | IARS, ileS | isoleucyl-tRNA synthetase [EC:6.1.1.5] | 0.00 | 0.14 |
| K11614 | yufL, malK | two-component system, CitB family, sensor histidine kinase MalK [EC:2.7.13.3] | 0.00 | 0.14 |
| K03527 | ispH, lytB | 4-hydroxy-3-methylbut-2-en-1-yl diphosphate reductase [EC:1.17.7.4] | 0.00 | 0.14 |
| K01804 | araA | L-arabinose isomerase [EC:5.3.1.4] | 0.00 | 0.14 |
| K08984 | yjdF | putative membrane protein | 0.00 | 0.14 |
| K02013 | ABC.FEV.A | iron complex transport system ATP-binding protein [EC:7.2.2.-] | 0.00 | 0.15 |
| K03544 | clpX, CLPX | ATP-dependent Clp protease ATP-binding subunit ClpX | 0.00 | 0.15 |
| K02111 | ATPF1A, atpA | F-type H+/Na+-transporting ATPase subunit alpha [EC:7.1.2.2 7.2.2.1] | 0.00 | 0.15 |
| K01945 | purD | phosphoribosylamine---glycine ligase [EC:6.3.4.13] | 0.00 | 0.15 |
| K01609 | trpC | indole-3-glycerol phosphate synthase [EC:4.1.1.48] | 0.00 | 0.16 |
| K03488 | licT, bglG | beta-glucoside operon transcriptional antiterminator | 0.00 | 0.16 |
| K01625 | eda | 2-dehydro-3-deoxyphosphogluconate aldolase / (4S)-4-hydroxy-2-oxoglutarate aldolase [EC:4.1.2.14 4.1.3.42] | 0.00 | 0.16 |
| K03545 | tig | trigger factor | 0.00 | 0.16 |
| K02879 | RP-L17, MRPL17 | large subunit ribosomal protein L17 | 0.00 | 0.16 |
| K01465 | URA4, pyrC | dihydroorotase [EC:3.5.2.3] | 0.00 | 0.16 |
| K03321 | TC.SULP | sulfate permease, SulP family | 0.00 | 0.17 |
| K00895 | pfp, PFP | diphosphate-dependent phosphofructokinase [EC:2.7.1.90] | 0.00 | 0.17 |
| K00874 | kdgK | 2-dehydro-3-deoxygluconokinase [EC:2.7.1.45] | 0.00 | 0.17 |
| K11381 | bkdA | 2-oxoisovalerate dehydrogenase E1 component [EC:1.2.4.4] | 0.00 | 0.17 |
| K01812 | uxaC | glucuronate isomerase [EC:5.3.1.12] | 0.00 | 0.17 |
| K12992 | rfbN | rhamnosyltransferase [EC:2.4.1.-] | 0.00 | 0.17 |
| K02933 | RP-L6, MRPL6, rplF | large subunit ribosomal protein L6 | 0.00 | 0.18 |
| K13919 | pduD | propanediol dehydratase medium subunit [EC:4.2.1.28] | 0.00 | 0.18 |
| K03648 | UNG, UDG | uracil-DNA glycosylase [EC:3.2.2.27] | 0.00 | 0.18 |
| K06199 | crcB, FEX | fluoride exporter | 0.00 | 0.18 |
| K03811 | pnuC | nicotinamide mononucleotide transporter | 0.00 | 0.18 |
| K02188 | cbiD | cobalt-precorrin-5B (C1)-methyltransferase [EC:2.1.1.195] | 0.00 | 0.18 |
| K02109 | ATPF0B, atpF | F-type H+-transporting ATPase subunit b | 0.00 | 0.18 |
| K02913 | RP-L33, MRPL33 | large subunit ribosomal protein L33 | 0.00 | 0.18 |
| K03547 | sbcD, mre11 | DNA repair protein SbcD/Mre11 | 0.00 | 0.18 |
| K02314 | dnaB | replicative DNA helicase [EC:3.6.4.12] | 0.00 | 0.19 |
| K03671 | trxA | thioredoxin 1 | 0.00 | 0.19 |
| K00100 | bdhAB | butanol dehydrogenase [EC:1.1.1.-] | 0.00 | 0.19 |
| K03546 | sbcC, rad50 | DNA repair protein SbcC/Rad50 | 0.00 | 0.19 |
| K00008 | SORD, gutB | L-iditol 2-dehydrogenase [EC:1.1.1.14] | 0.00 | 0.19 |
| K03738 | aor | aldehyde:ferredoxin oxidoreductase [EC:1.2.7.5] | 0.00 | 0.19 |
| K04844 | ycjT | hypothetical glycosyl hydrolase [EC:3.2.1.-] | 0.00 | 0.20 |
| K03149 | thiG | thiazole synthase [EC:2.8.1.10] | 0.00 | 0.20 |
| K01224 | E3.2.1.89 | arabinogalactan endo-1,4-beta-galactosidase [EC:3.2.1.89] | 0.00 | 0.20 |
| K00615 | tktA, tktB | transketolase [EC:2.2.1.1] | 0.00 | 0.21 |
| K07284 | srtA | sortase A [EC:3.4.22.70] | 0.00 | 0.21 |
| K01652 | ilvB, ilvG, ilvI | acetolactate synthase I/II/III large subunit [EC:2.2.1.6] | 0.00 | 0.21 |
| K00067 | rfbD, rmlD | dTDP-4-dehydrorhamnose reductase [EC:1.1.1.133] | 0.00 | 0.21 |
| K00789 | metK | S-adenosylmethionine synthetase [EC:2.5.1.6] | 0.00 | 0.21 |
| K10440 | rbsC | ribose transport system permease protein | 0.00 | 0.21 |
| K00854 | xylB, XYLB | xylulokinase [EC:2.7.1.17] | 0.00 | 0.21 |
| K01886 | QARS, glnS | glutaminyl-tRNA synthetase [EC:6.1.1.18] | 0.00 | 0.21 |
| K00005 | gldA | glycerol dehydrogenase [EC:1.1.1.6] | 0.00 | 0.21 |
| K01928 | murE | UDP-N-acetylmuramoyl-L-alanyl-D-glutamate--2,6-diaminopimelate ligase [EC:6.3.2.13] | 0.00 | 0.21 |
| K12240 | pchF | pyochelin synthetase | 0.00 | 0.21 |
| K00926 | arcC | carbamate kinase [EC:2.7.2.2] | 0.00 | 0.21 |
| K02113 | ATPF1D, atpH | F-type H+-transporting ATPase subunit delta | 0.00 | 0.21 |
| K00925 | ackA | acetate kinase [EC:2.7.2.1] | 0.00 | 0.21 |
| K00113 | glpC | glycerol-3-phosphate dehydrogenase subunit C | 0.00 | 0.22 |
| K13694 | mepS, spr | murein DD-endopeptidase / murein LD-carboxypeptidase [EC:3.4.-.- 3.4.17.13] | 0.00 | 0.22 |
| K02232 | cobQ, cbiP | adenosylcobyric acid synthase [EC:6.3.5.10] | 0.00 | 0.22 |
| K08221 | yitG, ymfD, yfmO | MFS transporter, ACDE family, multidrug resistance protein | 0.00 | 0.23 |
| K01610 | pckA | phosphoenolpyruvate carboxykinase (ATP) [EC:4.1.1.49] | 0.00 | 0.23 |
| K12999 | rgpI | glucosyltransferase [EC:2.4.1.-] | 0.00 | 0.23 |
| K02355 | fusA, GFM, EFG | elongation factor G | 0.00 | 0.23 |
| K01659 | prpC | 2-methylcitrate synthase [EC:2.3.3.5] | 0.00 | 0.23 |
| K14441 | rimO | ribosomal protein S12 methylthiotransferase [EC:2.8.4.4] | 0.00 | 0.23 |
| K01818 | fucI | L-fucose/D-arabinose isomerase [EC:5.3.1.25 5.3.1.3] | 0.00 | 0.24 |
| K04087 | hflC | membrane protease subunit HflC [EC:3.4.-.-] | 0.00 | 0.24 |
| K02745 | PTS-Aga-EIIB, agaV | PTS system, N-acetylgalactosamine-specific IIB component [EC:2.7.1.-] | 0.00 | 0.24 |
| K03641 | tolB | TolB protein | 0.00 | 0.24 |
| K10708 | frlB | fructoselysine 6-phosphate deglycase [EC:3.5.-.-] | 0.00 | 0.24 |
| K13920 | pduE | propanediol dehydratase small subunit [EC:4.2.1.28] | 0.00 | 0.24 |
| K00927 | PGK, pgk | phosphoglycerate kinase [EC:2.7.2.3] | 0.00 | 0.24 |
| K07454 | K07454 | putative restriction endonuclease | 0.00 | 0.24 |
| K03573 | mutH | DNA mismatch repair protein MutH | 0.00 | 0.25 |
| K06871 | K06871 | uncharacterized protein | 0.00 | 0.25 |
| K01443 | nagA, AMDHD2 | N-acetylglucosamine-6-phosphate deacetylase [EC:3.5.1.25] | 0.00 | 0.25 |
